# Physical Activity and Favorable Adiposity Genetic Liability Reduce the Risk of Hypertension Among High Body Mass Individuals

**DOI:** 10.1161/JAHA.124.040701

**Published:** 2025-10-23

**Authors:** Chukwueloka Hezekiah, Raha Pazoki

**Affiliations:** ^1^ Cardiovascular and Metabolic Research Group, Department of Biosciences, College of Health, Medicine and Life Sciences Brunel University London London United Kingdom; ^2^ Department of Public Health, Children’s, Learning Disability and Mental Health Nursing, Faculty of Health, Science, Social Care and Education Kingston University Surrey United Kingdom; ^3^ Department of Epidemiology and Biostatistics, School of Public Health Imperial College London London United Kingdom

**Keywords:** body mass, favorable adiposity, genetic liability, hypertension, physical activity, Cardiovascular Disease, Exercise, Epidemiology, Precision Medicine

## Abstract

**Background:**

Hypertension is a global health issue, and the risk factors include genetics, physical inactivity, and excess body fat (adiposity). Genetic predisposition to adiposity generally increases risk of hypertension. Several genetic variants have been identified to increase adiposity but unexpectedly reduce hypertension (favorable adiposity genes). Here, we tested the effect of these genetic variants on risk of hypertension in European ancestry participants under various scenarios of physical activity and body mass index.

**Methods:**

Favorable adiposity genetic liability was estimated using previously identified genetic variants and their effect sizes. The study analyzed data from 210 290 unrelated participants in the UK Biobank. Logistic regression was used to examine the association between this genetic liability and hypertension (defined as systolic blood pressure ≥140 mm Hg, diastolic blood pressure ≥90 mm Hg, or the use of antihypertensive medications). The analyses were conducted separately based on physical activity status (physically active and inactive) within low and high body mass groups.

**Results:**

Individuals with high body mass, could reduce their risk of hypertension by up to 13% depending on the favorable adiposity genetic liability and physical activity status (*P*_adjusted_=5.44×10^−7^). In high body mass individuals, physical activity alone contributes to 5% to 7% reduction in risk of hypertension.

**Conclusions:**

The study provides evidence that the protective effect of favorable adiposity on hypertension risk varies according to body mass composition and physical activity status.

**Nonstandard Abbreviation and Acronym**
UKB: UK Biobank

**Clinical Perspective**

**What Is New?**

- Favorable adiposity genetic liability can protect against stage 2 hypertension, but this protection depends on the individual's body mass index.
- For individuals with a high body mass index, physical activity offers additional protection against stage 2 hypertension, regardless of the individual's genetic liability.

**What Are the Clinical Implications?**

- The findings can help in developing more effective, personalized approaches to hypertension management, focusing on both genetic and lifestyle factors.

Hypertension is a significant global health concern affecting about 1.3 billion adults globally.^1^ Several risk factors for hypertension exist, including obesity indicated by high body mass^2^ as well as adiposity indicated by excess body fat.^3^ A large body of evidence exist emphasizing that excess body mass^4^, ^5^, ^6^ and total body fat mass percentage^3^ increased risk of hypertension.

For over a decade, genome‐wide association studies in the general population have identified common alleles associated with both obesity^7^, ^8^ and adiposity.^9^ The genetic liability that is driven by these genetic variants increase the risk of hypertension.^10^, ^11^ Recently, 36 genetic adiposity variants, associated with body fat percentage, have been identified as favorable adiposity variants.^12^ Favorable adiposity phenotype is defined as carrying a low risk of cardiometabolic disorders while carrying a large body fat percentage.^12^ Accumulating evidence from genetic studies suggests that individuals with favorable adiposity genes have lower risk of hypertension than individuals with unfavorable adiposity genes.^12^, ^13^ For example, Yaghootkar and colleagues^14^ reported an association between favorable adiposity alleles and a higher fat mass, a lower systolic and diastolic blood pressure, and a lower risk of hypertension.^14^ Individuals carrying favorable adiposity variants exhibit a healthy metabolic profile, reducing their risk of developing several conditions, including hypertension.^13^, ^14^ Ahmed and colleagues found that the favorable adiposity genetic liability is associated with a healthy metabolic profile and a low systolic blood pressure (SBP) and diastolic blood pressure (DBP) in various ethnic groups including European people.^13^ These findings confirm the beneficial effect of favorable adiposity alleles in reducing the risk of hypertension.

It is known that lifestyle factors such as regular physical activity can help reduce adiposity and mitigate the risk of hypertension.^11^, ^15^ In our previous work, we observed that genetic underpinning of body mass index (BMI) increases the risk of hypertension and physical activity offsets this risk.^11^ Here, we aim to test the effect of favorable adiposity genetic liability on hypertension in presence and absence of physical activity and for various subgroups of BMI within European ancestry participants of the UK Biobank (UKB). The research question is that whether physical activity offsets the effect of favorable adiposity on hypertension and what the pattern of association is among individuals with high and low BMI. Such research is crucial to provide more insight into personalized lifestyle choices, enhance effectiveness of treatments, leading to better management of hypertension and overall health outcomes.

## METHODS

The data used in this work were obtained from the UKB (application ID 60549), a resource for public health research that could be accessed by application to the UKB. The authors declare that the data used in this study are available to qualified researchers upon request from the UKB via the Access Management System.

### Study Population

The UKB is a major prospective cohort study that started in 2006 to allow detailed investigations of genetic and nongenetic determinants of disease. Between 2006 and 2010 (the initial assessment), more than 500 000 individuals of mainly European ancestry aged 40 to 69 were recruited by the UKB.^16^ During the initial assessment in the UKB, a wide range of phenotypic information was gathered following informed consent. The data were collected through interviews and touchscreen questionnaires, including sociodemographic, lifestyle, and health‐related information. Participants also underwent physical and anthropometric measurements during this assessment. The participants provided saliva, urine, and blood samples for proteomic, genetic, and metabolomic analyses.^17^

### Ethical Approval

Our study was conducted in accordance with the principles outlined in the declaration of Helsinki.^18^ The Northwest Multi‐Centre Research Ethics Committee (11/NW/0382) granted the UKB ethics approval as a research tissue bank approval. Participants gave their informed consent, and the UKB handled ethical approval. Using approved data request application ID 60549, data for this study were obtained from the UKB. The College of Health, Medicine, and Life Sciences Research Ethics Committee at Brunel University London provided ethical approval for the current analysis to work on secondary data from the UKB (reference 27684‐LR‐Jan/2021‐29 901 1).

### Genotyping and Imputation

The UKB did the genotyping and imputation centrally. Comprehensive techniques used by the UKB are provided elsewhere.^19^, ^20^, ^21^ In summary, the participants' blood samples were collected at the assessment center, and the DNA was extracted and genotyped by the UKB using the UKB Axiom Array. Three reference panels were used for genotype imputation: the Haplotype Reference Consortium, UK10K, and 1000 Genomes phase 3. IMPUTE4 program was used to carry out the imputation. The UKB calculated the kinship coefficients and genetic principal components centrally. These helped them identify related individuals and account for population stratification.^19^

### Sample for Analysis and Exclusion Criteria

The current study was performed using a subset of unrelated European ancestry individuals from the UKB. Genetic data were available for 488 377 individuals, and after merging genetic and phenotype data, 486 976 individuals remained for analysis. The current study applied several exclusion criteria. In summary, we excluded participants who withdrew consent (n=321), first‐ or second‐degree relatives (n=26 102), participants of non‐European ancestry (n=27 121), sex mismatch (n=328), pregnant women or women unsure of pregnancy status at baseline (n=513), or did not declare their smoking status (n=221). Furthermore, we also excluded participants with missing data in their pack‐years of smoking (n=66 062), current or previous smokers for whom zero pack‐years of smoking was calculated (n=1049), unsure about their dietary intake of fish, meat, fruits, or vegetables (n=12 919) and participants on cholesterol‐lowering medication (n=60 090). In addition, participants who did not declare drinking status (n=160), participants whose self‐reported ancestry did not match their genetic ancestry (n=44) and individuals with missing data in the main study covariates (n=82 077) were also excluded. A total of 210 290 participants remained for analysis (Figure S1).

### Blood Pressure and Definition of Hypertension

BP was measured centrally by the UKB. The details of the BPphenotype have been described previously.^11^ In brief, SBP and DBP were measured twice at the UKB assessment center using an automated sphygmomanometer (Omron HEM 7015‐T) or a manual sphygmomanometer where an automated BP reading could not be taken. This study used the mean of these readings obtained using the same device (automated or manual sphygmomanometer) to calculate the average BP (SBP and DBP). Where BP values obtained from different devices existed, we calculated average BP using values from those devices. For participants on BP‐lowering medication, we added 15 mm Hg to the SBP and 10 mm Hg to the DBP^22^ (n=89 524). We defined hypertension as stage 2 according to the American Heart Association guidelines,^23^ SBP ≥140 or DBP ≥90 mm Hg. We additionally defined the use of BP‐lowering medications as hypertension.

### Physical Activity Status

The details of the physical activity phenotype have been described previously.^11^ In summary, the UKB used a modified version of the short International Physical Activity Questionnaire to measure physical activity^24^ using a touchscreen questionnaire. They collected data on the frequency, intensity, and duration of walking and moderate and vigorous physical activity. Cassidy and colleagues^25^ calculated the metabolic equivalent of task (MET) minutes per week of physical activity using the International Physical Activity Questionnaire data processing guidelines,^26^ and the data were returned to the UKB. The current study used the returned data from Cassidy and colleagues^25^ to categorize participants into physically active (moderate activity ≥150 minutes per week or vigorous activity ≥75 MET minutes per week or summed MET minutes per week for all activities ≥600). We categorized participants who did not meet these criteria into a physically inactive group.

### Obesity Status

UKB determined the BMI values during the initial assessment center visit by dividing weight in kilograms (measured using the Tanita BC‐418 MA body composition analyzer) by height in meters (measured using a Seca 202 device). If either of these values were absent, the UKB omitted the BMI value for those participants.^16^ The current study used the BMI values to define the participants' obesity status. We classified BMI (kg/m^2^) using the World Health Organization's categories: underweight (<18.5), normal weight (≥18.5 to <25), overweight (≥25 to <30), and obese (≥30).^27^ In the present study, we categorized participants using the BMI values into (1) high body mass (overweight and obese) and (2) low body mass (normal weight and underweight).

### Assessment of Covariates

The UKB used a self‐reported questionnaire to assess participants' dietary intake. Using touchscreen multiple‐choice questions, participants selected their daily food consumption (vegetable, fruit, oily fish, and meat intake). Smoking status in the UKB was determined through self‐reported questions and categorized into current, past, and never smoking. Pack‐years of smoking were available for the sample. The pack‐years of smoking were calculated based on the number of cigarettes smoked per day, divided by the average pack‐size of 20, and multiplied by the number of years smoking.^28^ This current study defined smoking based on the values from smoking status and pack‐years of smoking. We defined smokers as current or previous smokers with any value in pack‐years. Never smokers were those who reported never smoking and had zero values in their pack‐years of smoking. We also included alcohol drinking status (current, past, and nondrinkers),^29^ low‐density lipoprotein cholesterol, high‐density lipoprotein cholesterol, and diabetes (defined as diabetes diagnosed by a doctor, or using insulin medication, or a record of serum level of hemoglobin A1c≥48 mmol/mol (6.5%), or glucose level≥7.0 mmol/dL)^30^ as additional covariates in the analysis. Detailed quality control and sample processing information for the UKB biomarker data has been published previously.^31^

### Favorable Adiposity Genetic Liability

We estimated favorable adiposity genetic liability based on a list of previously published single nucleotide polymorphisms (SNPs) associated with favorable adiposity from Martin and colleagues.^12^ We used the effect estimates of these SNPs on favorable adiposity as their weight in calculation of the genetic liability. Using LDlink,^32^ we assessed the linkage disequilibrium among the SNPs. As part of the SNP selection process in this study, we considered only SNPs that met the genome‐wide association studies significance threshold of *P*<5×10^−8^, and SNPs with minor allele frequency>0.01. We defined SNP pairs with R^2^>0.1 as correlated. Therefore, only SNPs that did not exhibit dependence on another SNP based on the linkage disequilibrium parameter of R^2^<0.1 were considered. The final number of SNPs available for estimating the genetic liability for favorable adiposity was 35 (Table S1). One SNP (rs555162510) that was not present in the 1000 Genomes Project data was removed. Using Plink v2.0^33^, ^34^ to calculate genetic liabilities, we multiplied the number of risk alleles each UKB participant carries on the favorable adiposity SNPs by their effect estimate. The products were summed up to generate the genetic liability, which was then standardized using the *base* package in R. The association results were therefore presented per SD increase in the genetic liability of favorable adiposity. The thresholds for genetic liability were based on tertiles of the population: low genetic liability ranged from −4.50 to −0.44, moderate genetic liability ranged from −0.44 to 0.42, and high genetic liability ranged from 0.42 to 4.38.

### Statistical Analysis

We conducted a cluster analysis using the K‐means algorithm to determine the participants' genetic distance.^35^ We standardized the first 40 genetic principal components for cluster analysis and used the K‐means algorithm to determine participants' genetic distance. The K‐means algorithm requires a parameter indicating the number of clusters (K).^36^ Based on the number of categories in the UKB self‐reported phenotypic variable ‘ethnic background,’ we assigned a K value of 7 (Table S2). We used a multidimensional scaling statistical technique to visualize the level of similarity between individual clusters in our data set. Participants were assigned to clusters (genetic ancestry) based on their genetic distances. We compared the self‐reported ancestry from the UKB phenotypic variable “ethnic background” with the genetically derived ancestry from the cluster analysis, focusing on the dominant clusters where this genetically derived ancestry was prevalent. We excluded 44 participants whose self‐reported ancestry did not match their genetically derived ancestry. The cluster analysis was performed using the *Mclust* and *Cluster* packages in R.

We compared the prevalence of hypertension within different categories of favorable adiposity genetic liability (moderate and high) against a reference group consisting of participants with low genetic liability. Using logistic regression, we examined the association between genetic liability and the odds of hypertension across high and low body mass groups. We additionally stratified our analysis by physical activity status and compared the results within each stratum (physically active or physically inactive) to assess the pattern of the association between genetic liability and hypertension across the physical activity and BMI strata. To exclude any potential effect of BP‐lowering medication on the findings, we performed a sensitivity analysis in which we repeated our analyses excluding participants on BP‐lowering medication from our primary analysis (n=26 409). Within the whole sample, an interaction test was performed to determine whether the effect of genetic liability on hypertension was modulated by physical activity.

To exclude bias due to any potential effect of favorable adiposity SNPs on physical activity, we conducted a single SNP analysis to assess the effect of each favorable adiposity SNP on physical activity. We assessed this effect across the entire population, as well as within the high and low body mass subpopulations. The primary objective was to investigate whether the protective effect of favorable adiposity genetic factors against hypertension is influenced indirectly through physical activity. If we identified SNPs significantly associated with physical activity, we performed a sensitivity analysis in which we excluded these SNPs to create a new favorable adiposity genetic liability. We then reexamined the association between favorable adiposity genetic liability and hypertension. To understand the combined effect of genetic liability and physical activity status on the prevalence of hypertension, we selected a reference group from participants with low genetic liability and physical inactivity. The odds of hypertension in all other combinations of genetic liability and physical activity was compared with this reference group.

To additionally quantify the pure effect of physical activity on the observed effects, we compared the modulatory effect of physical activity on the association of genetic liability and hypertension within subgroups with similar BMI and genetic liability.

Statistical significance was accepted if the type 1 error (*P* value) was <0.05. All statistical analyses were conducted in R v4.0.0.^37^ We used the Strengthening the Reporting of Observational Studies in Epidemiology cohort checklist when writing our report.^38^

## Sensitivity Analysis Using Accelerometer Data

We performed a sensitivity analysis using accelerometer data (Supplementary Method) because the accelerometer data were measured in 2013 to 2015 and not at the first visit (2006–2010) when the hypertension status for this study was assessed. Thus, this analysis should be interpreted with caution due to potential reverse causation. The details of this sensitivity analysis are provided in the supplemental materials section.^39^, ^40^, ^41^, ^42^

## RESULTS

This study included 210 290 UKB participants of European ancestry (Table 1). Within the whole sample studied, 132 278 had high body mass (BMI ≥25 kg/m^2^), and 78 012 had low body mass. Physically inactive participants had a higher BMI, with a mean of 28.2 kg/m^2^ (SD=5.33), compared with physically active participants, (mean BMI=26.7 kg/m^2^; SD=4.39). Among physically active participants 45.8% were hypertensive, which was less than physically inactive participants (47.4%; *P*<0.001).

**Table 1:** Baseline Characteristics of the Study Sample

Characteristics	Whole sample (n=210 290)			High body mass (n=132 278)			Low body mass (n=78 012)		
	Physically active (n=183 873)	Physically inactive (n=26 417)	*P* value for differences in the characteristic according to physical activity status*	Physically active (n=113 425)	Physically inactive (n=18 853)	*P* value for differences in the characteristic according to physical activity status*	Physically active (n=70 448)	Physically inactive (n=7564)	*P* value for differences in the characteristic according to physical activity status*
Age, y									
Mean±SD	55.5 (8.0)	54.9 (7.7)	<0.001	55.8 (8.0)	55.1 (7.7)	<0.001	55.1 (8.1)	54.3 (7.8)	<0.001
Median [min, max]	56.0 [38–73]	55.0 [40–70]		57.0 [39–73]	55.0 [40–70]		56.0 [38–70]	55.0 [40–70]	
Sex									
Female, n (%)	101 494 (55.2%)	14 777 (55.9%)	0.02	55 495 (48.9%)	9900 (52.5%)	<0.001	45 999 (65.3%)	4877 (64.5%)	0.16
Male, n (%)	82 379 (44.8%)	11 640 (44.1%)		57 930 (51.1%)	8953 (47.5%)		24 449 (34.7%)	2687 (35.5%)	
Body mass index, kg/m^2^									
Mean±SD	26.7 (4.39)	28.2 (5.33)	<0.001	29.2 (3.71)	30.4 (4.66)	<0.001	22.8 (1.65)	22.7 (1.72)	0.55
Median [min, max]	26.1 [12.1–66.2]	27.4 [15.2–65.0]		28.2 [25.0–66.2]	29.2 [25.0–65.0]		23.1 [12.1–25.0]	23.1 [15.2–25.0]	
Stage 2 hypertension cases†									
No, n (%)	99 695 (54.2%)	13 887 (52.6%)	<0.001	53 157 (46.9%)	8759 (46.5%)	0.31	46 538 (66.1%)	5128 (67.8%)	2.54×10^−3^
Yes, n (%)	84 178 (45.8%)	12 530 (47.4%)		60 268 (53.1%)	10 094 (53.5%)		23 910 (33.9%)	2436 (32.2%)	
Low‐density lipoprotein cholesterol, mmol/L									
Mean±SD	3.71 (0.8)	3.76 (0.8)	<0.001	3.81 (0.8)	3.83 (0.8)	0.081	3.55 (0.8)	3.60 (0.8)	<0.001
Median [min, max]	3.67 [0.80–9.74]	3.72 [0.80–7.48]		3.78 [0.80–9.74]	3.79 [0.97–7.45]		3.49 [0.89–9.04]	3.54 [0.80–7.48]	
High‐density lipoprotein cholesterol, mmol/L									
Mean±SD	1.49 (0.4)	1.40 (0.4)	<0.001	1.39 (0.3)	1.33 (0.3)	<0.001	1.64 (0.4)	1.57 (0.4)	<0.001
Median [min, max]	1.44 [0.23–4.40]	1.35 [0.45–4.04]		1.35 [0.35–4.09]	1.29 [0.45–3.60]		1.61 [0.23–4.40]	1.53 [0.48–4.04]	
Smoking status									
Nonsmoker, n (%)	122 589 (66.7%)	16 707 (63.2%)	<0.001	72 785 (64.2%)	11 628 (61.7%)	<0.001	49 804 (70.7%)	5079 (67.1%)	<0.001
Smoker, n (%)	61 284 (33.3%)	9710 (36.8%)		40 640 (35.8%)	7225 (38.3%)		20 644 (29.3%)	2485 (32.9%)	
Systolic blood pressure (mm Hg)									
Mean±SD	139 (19.9)	139 (19.9)	0.39	142 (19.5)	141 (19.4)	<0.001	133 (19.5)	132 (19.8)	<0.001
Median [min, max]	137 [80–268]	136 [72–253]		140 [85–268]	139 [87–253]		131 [80–237]	129 [72–229]	
Diastolic blood pressure (mm Hg)									
Mean±SD	83.2 (11.0)	84.5 (11.4)	<0.001	85.7 (10.7)	86.5 (11.0)	<0.001	79.3 (10.3)	79.5 (10.7)	0.10
Median [min, max]	82.5 [42–148]	83.5 [46–141]		85.0 [46–148]	85.5 [49–141]		78.5 [42–134]	78.5 [46–130]	
Alcohol status									
Never, n (%)	5510 (3.0%)	977 (3.7%)	<0.001	3366 (3.0%)	706 (3.7%)	<0.001	2144 (3.0%)	271 (3.6%)	<0.001
Previous, n (%)	5496 (3.0%)	1064 (4.0%)		3451 (3.0%)	753 (4.0%)		2045 (2.9%)	311 (4.1%)	
Current, n (%)	172 867 (94.0%)	24 376 (92.3%)		106 608 (94.0%)	17 394 (92.3%)		66 259 (94.1%)	6982 (92.3%)	
Daily fruit and vegetable intake									
Mean±SD	8.1 (4.52)	6.6 (4.01)	<0.001	8.0 (4.46)	6.6 (3.99)	<0.001	8.3 (4.61)	6.6 (4.06)	<0.001
Median [min, max]	7.0 [0–130]	6.0 [0–80]		7.0 [0–110]	6.0 [0–72]		7.0 [0–130]	6.0 [0–80.0]	
Oily fish intake									
Mean±SD	1.7 (0.91)	1.5 (0.89)	<0.001	1.6 (0.92)	1.4 (0.90)	<0.001	1.7 (0.91)	1.5 (0.88)	<0.001
Median [min, max]	2.0 [0–5]	1.0 [0–5]		2.0 [0–5]	1.0 [0–5]		2.0 [0–5]	1.0 [0–5]	
Meat intake									
Mean±SD	7.8 (2.81)	8.0 (2.67)	<0.001	8.1 (2.65)	8.2 (2.57)	0.02	7.3 (2.98)	7.5 (2.84)	<0.001
Median [min, max]	8.0 [0–25]	8.0 [0–20]		8.0 [0–25]	8.0 [0–20]		8.0 [0–25]	8.0 [0–17]	
Diabetes									
No, n (%)	178 754 (97.2%)	25 302 (95.8%)	<0.001	109 426 (96.5%)	17 883 (94.9%)		69 328 (98.4%)	7419 (98.1%)	
Yes, n (%)	5119 (2.8%)	1115 (4.2%)		3999 (3.5%)	970 (5.1%)	<0.001	1120 (1.6%)	145 (1.9%)	0.04

* *P* value is provided for the difference in the obesity/physical activity status. Statistical analyses were performed using the chi‐square test for categorical variables and ANOVA for numerical variables.

^†^ Systolic blood pressure>140 mm Hg or diastolic blood pressure>90 mm Hg.

Within the high body mass group, 85.8% (n=113 425) participants were physically active. We observed no significant difference in the prevalence of stage 2 hypertension between physically active versus physically inactive participants with high‐body mass (*P*=0.31).

Among participants with low‐body mass, 90.3% (n=70 448) were physically active. Within this group, we observed no difference in the BMI between physically active and physically inactive participants (*P*=0.55). Among physically active participants with low body mass, 33.9% were hypertensive, which was more than physically inactive participants (32.2%; *P*=2.54×10^−3^).

### The Effect of Genetic Liability on Hypertension Stratified for Body Mass

Among participants with high body mass, the odds of hypertension were significantly reduced depending on levels of genetic liability (adjusted odds ratio [aOR] _moderate versus low genetic liability_, 0.96 [95% CI, 0.93–0.99]; *P*=4.92×10^−3^; aOR _high versus low genetic liability,_ 0.93 [95% CI, 0.91–0.96]; *P*=3.05×10^−7^) (Table 2). Similar results were observed within the low body mass sample (aOR _moderate versus low genetic liability,_ 0.95 [95% CI, 0.92–0.99]; *P*=1.22×10^−2^; aOR _high versus low genetic liability,_ 0.93 [95% CI, 0.89–0.96]; *P*=8.61×10^−5^) (Table 2).

**Table 2:** The Effect of Genetic Liability on Hypertension Stratified for Body Mass

Obesity status	FA genetic liability categories	Hypertensive (n)	Nonhypertensive (n)	% hypertensive	Unadjusted			Minimally adjusted*			Adjusted ^†^		
					Odds ratio^‡^	95% CI^§^	*P* value for odds ratio^‖^	Odds ratio^‡^	95% CI^§^	*P* value for odds ratio^‖^	Odds ratio^‡^	95% CI^§^	*P* value for odds ratio^‖^
High body mass	Low	23 409	19 790	54.19	1 (reference)			1 (reference)			1 (reference)		
	Moderate	23 350	20 634	53.09	0.96	0.93–0.98	1.12×10^−3^	0.95	0.93–0.98	4.89×10^−4^	0.96	0.93–0.99	4.92×10^−3^
	High	23 603	21 492	52.34	0.93	0.90–0.95	3.77×10^−8^	0.91	0.89–0.94	1.39×10^−10^	0.93	0.91–0.96	3.05×10^−7^
Low body mass	Low	9299	17 665	34.49	1 (reference)			1 (reference)			1 (reference)		
	Moderate	8745	17 309	33.56	0.96	0.93–0.99	2.51×10^−2^	0.96	0.92–0.99	1.93×10^−2^	0.95	0.92–0.99	1.22×10^−2^
	High	8302	16 692	33.22	0.95	0.91–0.98	2.23×10^−3^	0.93	0.89–0.97	2.43×10^−4^	0.93	0.89–0.96	8.61×10^−5^

Odds ratios show the odds of prevalent stage 2 hypertension for participants belonging to each combination compared with the reference group (low genetic liability). Physical activity was defined as METs for moderate activity ≥150 OR METs for vigorous activity ≥75 OR summed METs for all activity ≥600. FA indicates favorable adiposity; and MET, metabolic equivalent task. *Adjusted for age and sex. ^†^Adjusted for age, sex, smoking status, alcohol status, meat and fish intake, fruit and vegetable intake, low‐density lipoprotein cholesterol, high‐density lipoprotein cholesterol, and diabetes (diagnosed by doctor OR using insulin medication OR glucose ≥7.0 mmol/L OR hemoglobin A1C ≥ 48 mmol/mol (6.5%). ^‡,§,‖^Odds ratio, 95% CI, and *P* value for odds ratio are provided for the joint association between favorable adiposity genetic liability and physical activity status on the odds of hypertension. The values are derived from logistic regression models.

### The Effect of Genetic Liability on Hypertension Stratified for Body Mass and Physical Activity Status

Table 3 shows the modulatory effect of physical activity on the association between favorable adiposity genetic liability and hypertension stratified by BMI. Within the high body mass sample (Table 3), the favorable adiposity genetic liability reduced the odds of hypertension in all subgroups including within the whole sample (aOR, 0.97 [95% CI, 0.96–0.98]; *P*=8.96×10^−8^), within the physically active subgroup (aOR, 0.97 [95% CI, 0.96–0.98]; *P*=6.67×10^−7^), as well as within the physically inactive subgroups (aOR, 0.97 [95% CI, 0.94–1.00]; *P*=4.12×10^−2^). Among the low body mass sample, similar results were observed for the whole sample (aOR, 0.96 [95% CI, 0.95–0.98]; *P*=7.03×10^−6^) and the physically active group (aOR, 0.96 [95% CI, 0.95–0.98]; *P*=2.09×10^−5^) but not for the physically inactive subgroup.

**Table 3:** The Effect of Genetic Liability on Hypertension Stratified for Body Mass and Physical Activity Status

Main analysis (n=210 290)																									
Genetic liability	High body mass and Low body mass					High body mass (n=132 278)										Low body mass (n=78 012)									
	Whole population (n=210 290)					Whole population (n=132 278)				Physically active (n=13 425)			Physically inactive (n=18 853)			Whole population (n=78 012)				Physically active (n=70 448)			Physically inactive (n=7564)		
	Odds ratio	95% CI	*P* value for odds ratio	Interaction *P* value*	Interaction *P* value*	Odds ratio	95% CI	*P* value for odds ratio	Interaction *P* value*	Odds ratio	95% CI	*P* value for odds ratio	Odds ratio	95% CI	*P* value for odds ratio	Odds ratio	95% CI	*P* value for odds ratio	Interaction *P* value*	Odds ratio	95% CI	*P* value for odds ratio	Odds ratio	95% CI	*P* value for odds ratio
Unadjusted odds ratios																									
Favorable adiposity	0.98	0.97–0.99	2.09×10^−6^	0.17	1.31×10^−3^	0.97	0.96–0.98	4.47×10^−9^	0.69	0.97	0.96–0.98	1.15×10^−8^	0.98	0.95–1.01	0.12	0.97	0.96–0.99	2.86×10^−4^	0.19	0.97	0.96–0.99	2.66×10^−4^	0.99	0.94–1.04	0.59
Minimally adjusted odds ratios^†^																									
Favorable adiposity	0.97	0.96–0.98	4.83×10^−10^	0.61	2.17×10^−3^	0.96	0.95–0.97	9.33×10^−12^	0.80	0.96	0.95–0.97	2.77×10^−10^	0.96	0.93–0.99	8.82×10^−3^	0.97	0.95–0.98	1.88×10^−5^	0.45	0.97	0.95–0.98	3.30×10^−5^	0.97	0.92–1.02	0.28
Adjusted odds ratios^‡^																									
Favorable adiposity	0.98	0.97–0.99	2.77×10^−4^	0.53	5.41×10^−3^	0.97	0.96–0.98	8.96×10^−8^	0.85	0.97	0.96–0.98	6.67×10^−7^	0.97	0.94–1.00	4.12×10^−2^	0.96	0.95–0.98	7.03×10^−6^	0.49	0.96	0.95–0.98	2.09×10^−5^	0.96	0.91–1.01	0.11

Sensitivity analysis: Excluded 24 205 participants on blood pressure‐lowering medication (n=186 085)																									
Genetic liability	High body mass and low body mass					High body mass (n=113 241)										Low body mass (n=67 722)									
	Whole population (n=186 085)					Whole population (n=113 241)				Physically active (n=97 515)			Physically inactive (n=15 726)			Whole population (n=72 844)				Physically active (n=65 865)			Physically inactive (n=6979)		
Unadjusted odds ratios																									
Favorable adiposity	0.99	0.98–1.00	4.51×10^−3^	0.14	1.38×10^−3^	0.98	0.96–0.99	3.90×10^−5^	0.72	0.97	0.96–0.99	6.99×10^−5^	0.98	0.95–1.01	0.28	0.98	0.97–1.00	2.73×10^−2^	0.07	0.98	0.96–1.00	1.58×10^−2^	1.01	0.96–1.06	0.78
Minimally adjusted odds ratios†																									
Favorable adiposity	0.98	0.97–0.99	1.76×10^−5^	0.45	3.05×10^−3^	0.97	0.96–0.98	5.57×10^−7^	0.88	0.97	0.96–0.98	4.65×10^−6^	0.97	0.94–1.00	5.05×10^−2^	0.98	0.96–0.99	3.17×10^−3^	0.20	0.97	0.96–0.99	2.48×10^−3^	0.99	0.94–1.05	0.81
Adjusted odds ratios‡																									
Favorable adiposity	0.99	0.98–1.00	1.80×10^−2^	0.32	5.60×10^−3^	0.98	0.96–0.99	6.07×10^−5^	1.00	0.98	0.96–0.99	2.10×10^−4^	0.98	0.94–1.01	0.14	0.97	0.96–0.99	1.38×10^−3^	0.20	0.97	0.95–0.99	1.49×10^−3^	0.98	0.93–1.04	0.47

Odds ratios were presented for the effect of physical activity on the genetic liability of favorable adiposity on hypertension according to obesity status. Two groups of physical activity were considered (physically active vs physically inactive). Physical activity was defined as METs for moderate activity ≥150 OR METs for vigorous activity ≥75 OR summed METs for all activity ≥600. MET indicates metabolic equivalent task.

* *P* value is provided for the interaction between favorable adiposity genetic liability and physical activity on an additive scale.

^†^ Adjusted for age and sex.

^‡^ Adjusted for age, sex, smoking status, alcohol status, meat and fish intake, fruit and vegetable intake, low‐density lipoprotein cholesterol, high‐density lipoprotein cholesterol, and diabetes (diagnosed by doctor OR using insulin medication OR glucose ≥7.0 mmol/L OR hemoglobin A1C≥48 mmol/mol (6.5%).

^§^ *P* value is also provided for the interaction between favorable adiposity genetic liability and BMI.

As a sensitivity analysis, participants taking BP‐lowering medication were excluded. Among individuals with high body mass, the results stayed similar for the whole sample and the physically active group but not for the physically inactive subgroup (*P*=0.14; Table 3). Among individuals with low body mass, similar results were also observed both within the whole sample (aOR, 0.98 [95% CI, 0.96–0.99]; *P*=6.07×10^−5^) and within the physically active sample (aOR, 0.98 [95% CI, 0.96–0.99]; *P*=2.10×10^−4^; Table 3).

### The Effect of Favorable Adiposity SNPs on Physical Activity

We investigated whether the protective effect of favorable adiposity SNPs on BMI and therefore hypertension could potentially be driven by physical activity. To explore this, we examined the impact of all favorable adiposity‐related SNPs on physical activity (Table S3) and BMI (Table S4). Of the 35 favorable adiposity SNPs used in estimating the favorable adiposity genetic liability, rs987469 was associated with reduced physical activity within the whole sample (adjusted β −12.04 per copy C allele, [95% CI, −23.22 to −0.87]; *P*=0.03) and within the high body mass sample (adjusted β −18.01 per copy C allele, [95% CI, −32.10 to −3.91]; *P*=0.01). Additionally, rs12369179 (adjusted β −14.90 per copy C allele, [95% CI, −28.81 to −0.99]; *P*=0.04) and rs573454216 (adjusted β −15.99 per copy G allele, [95% CI, −30.11 to −1.88]; *P*=0.03) were associated with reduced physical activity within the high body mass sample. A statistically significant association was also observed in the relationship between rs4684847 and increased physical activity within the whole sample (adjusted β 15.89 per copy C allele, [95% CI, 4.85–26.93]; *P*=4.79×10^−3^) and within the high body mass sample (adjusted β 18.77 per copy C allele, [95% CI, 4.88–32.65]; *P*=8.06×10^−3^) (Table S3). For rs12369179, rs4684847, and rs987469 the allele that reduced physical activity increased BMI (Table S4).

We observed that the genetic liability was not associated with physical activity within the whole sample or the low body mass subgroups. However, the genetic liability was associated with reduced physical activity within the high body mass sample (adjusted β, −16.18 [95% CI, −30.40 to −1.96]; *P*=0.03). After excluding the physical activity‐related SNPs identified in our previous step (rs12369179, rs4684847, rs987469, and rs573454216), no association between genetic liability and physical activity was observed within the whole sample or any of the subgroups (Table S3).

We repeated the main association analyses between genetic liability and hypertension (stratified by physical activity and BMI) excluding the 4 physical activity‐related SNPs and observed no remarkable changes in the findings expect in the physically inactive subgroup of the high body mass sample (*P*=0.11; Table S5).

### Joint Effects of Genetic Liability and Physical Activity on the Odds of Hypertension Stratified by the BMI (Excluding Physical Activity‐Related SNPs)

Stratified by BMI, the joint effect of the genetic liability and physical activity on the odds of hypertension was compared with a reference group that included individuals who had a combination of low genetic liability and low physical activity as both of these factors are not optimal for odds of hypertension (Table 4). Among high body mass participants, combinations of genetic liability and being physically active were associated with lower odds of hypertension compared with the reference group (aOR _low genetic liability, physically active,_ 0.93 [95% CI, 0.880.98]; *P*=7.84×10^−3^; aOR _moderate genetic liability, physically active,_ 0.90 [95% CI, 0.85–0.95]; *P*=1.20×10^−4^; aOR _high genetic liability, physically active,_ 0.87 [95% CI, 0.82–0.92]; *P*=5.44×10^−7^; Table 4; Table S6). Among low body mass participants, no association was observed between any combinations of genetic liability and physical activity status with the odds of hypertension (Table 4; Table S6).

**Table 4:** Joint Effects of Genetic Liability and Physical Activity on the Odds of Hypertension Stratified by BMI (Excluding Physical Activity‐Related SNPs*)

Obesity status	FA genetic liability^*^ categories	Physical activity status	Hypertensive (n)	Nonhypertensive (n)	% hypertensive	Unadjusted			Minimally adjusted^†^			Adjusted ^‡^		
						Odds ratio^║^	95% CI^¶^	*P* value for odds ratio*	Odds ratio^‖^	95% CI^¶^	*P* value for odds ratio^#^	Odds ratio^║^	95% CI^¶^	*P* value for odds ratio^#^
High body mass (n=132 278)	Low	Physically inactive	3354	2821	54.32	1 (reference)			1 (reference)			1 (reference)		
	Low	Physically active	20 386	17 340	54.04	0.99	0.94–1.04	0.68	0.91	0.86–0.96	6.14×10^−4^	0.93	0.88–0.98	7.84×10^−3^
	Moderate	Physically inactive	3302	2896	53.28	0.96	0.89–1.03	0.25	0.93	0.87–1.00	5.74×10^−2^	0.94	0.88–1.02	0.13
	Moderate	Physically active	19 914	17 591	53.10	0.95	0.90–1.01	7.53×10^−2^	0.87	0.82–0.92	8.81×10^−4^	0.90	0.85–0.95	1.20×10^−4^
	High	Physically inactive	3438	3042	53.06	0.95	0.89–1.02	0.16	0.91	0.85–0.98	1.19×10^−2^	0.93	0.86–1.00	4.56×10^−2^
	High	Physically active	19 968	18 226	52.28	0.92	0.87–0.97	2.97×10^−3^	0.83	0.79–0.88	1.96×10^−10^	0.87	0.82–0.92	5.44×10^−7^
Low body mass (n=78 012)	Low	Physically inactive	866	1836	32.05	1 (reference)			1 (reference)			1 (reference)		
	Low	Physically active	8435	15 985	34.54	1.12	1.03–1.22	9.67×10^−3^	1.03	0.94–1.12	0.55	1.03	0.94–1.13	0.52
	Moderate	Physically inactive	799	1631	32.88	1.04	0.92–1.17	0.53	1.00	0.89–1.13	0.99	0.98	0.87–1.11	0.79
	Moderate	Physically active	7980	15 459	34.05	1.09	1.01–1.19	3.80×10^−2^	1.00	0.91–1.09	0.98	1.00	0.91–1.09	0.97
	High	Physically inactive	771	1661	31.70	0.98	0.88–1.11	0.79	0.94	0.83–1.06	0.30	0.93	0.82–1.05	0.23
	High	Physically active	7495	15 094	33.18	1.05	0.97–1.15	0.24	0.96	0.88–1.05	0.34	0.96	0.87–1.05	0.35

Odds ratios show the odds of prevalent stage 2 hypertension for participants belonging to each combination compared with the reference group (low genetic liability combined with physically inactive). Physical activity was defined as METs for moderate activity ≥150 OR METs for vigorous activity ≥75 OR summed METs for all activity ≥600. FA indicates favorable adiposity; and MET, metabolic equivalent task. *Estimated genetic liability without rs12369179, rs4684847, rs987469 and rs573454216, ^†^Adjusted for age and sex. ^‡^Adjusted for age, sex, smoking status, alcohol status, meat and fish intake, fruit and vegetable intake, low‐density lipoprotein cholesterol, high‐density lipoprotein cholesterol, and diabetes (diagnosed by doctor OR using insulin medication OR glucose ≥7.0 mmol/L OR hemoglobin A1C ≥ 48 mmol/mol (6.5%). ^║,¶,#^Odds ratio, 95% CI and *P* value for odds ratio, are provided for the joint association between favorable adiposity genetic liability and physical activity status on the odds of hypertension. The values are derived from logistic regression models.

We also observed that physical activity reduced the odds of hypertension beyond genetic liability among high body mass individuals. In brief, the effect of the combination of low genetic liability and physical activity was significantly lower than the combination of low genetic liability and physical inactivity (OR, 0.93 [95% CI, 0.88–0.98]; *P*=1.21×10^−2^). Similar results were observed for the combination of moderate genetic liability and physical activity versus the combination of moderate genetic liability and physical inactivity (OR, 0.95 [95% CI, 0.89–1.00]; *P*=5.34×10^−2^) as well as the combination of high genetic liability and physical activity versus the combination of high genetic liability and physical inactivity (OR, 0.93 [95% CI, 0.88–0.98]; *P*=1.15×10^−2^) (Table 5). In contrast, within the low body mass participants no association was found in terms of physical activity reducing the odds of hypertension across any level of genetic liability (Table 5).

**Table 5:** Influence of Physical Activity on the Odds of Hypertension Across Different Categories of Favorable Adiposity Genetic Liability

Obesity status	FA genetic liability categories	Physical activity status	Genetic liability includes all SNPs			Sensitivity analysis excluding physical activity SNPs		
			Adjusted*			Adjusted*		
			Odds ratio^†^	95% CI^‡^	*P* value^§^	Odds ratio^†^	95% CI^‡^	*P* value^§^
High body mass (n=132 278)	Low	Physically inactive	1 (reference)	…	…	1 (reference)	…	…
	Low	Physically active	0.95	0.89–1.00	6.26×10^−2^	0.93	0.88–0.98	1.21×10^−2^
	Moderate	Physically inactive	1 (reference)	…	…	1 (reference)	…	…
	Moderate	Physically active	0.91	0.86–0.97	1.52×10^−3^	0.95	0.89–1.00	5.34×x10^−2^
	High	Physically inactive	1 (reference)	…	…	1 (reference)	…	…
	High	Physically active	0.95	0.89–1.00	4.61×10^−2^	0.93	0.88–0.98	1.15×10^−2^
Low body mass (n=78 012)	Low	Physically inactive	1 (reference)	…	…	1 (reference)	…	…
	Low	Physically active	1.06	0.97–1.16	0.21	1.04	0.95–1.13	0.46
	Moderate	Physically inactive	1 (reference)	…	…	1 (reference)	…	…
	Moderate	Physically active	1.03	0.93–1.13	0.60	1.02	0.93–1.12	0.71
	High	Physically inactive	1 (reference)	…	……	1 (reference)	…	…
	High	Physically active	1.00	0.91–1.10	0.95	1.03	0.93–1.13	0.57

Odds ratios show the odds of prevalent stage 2 hypertension for participants belonging to each combination compared with the reference group (comprising similar genetic liability combined with physically inactive). Physical activity was defined as METs for moderate activity ≥150 OR METs for vigorous activity ≥75 OR summed METs for all activity ≥600. FA indicates favorable adiposity; and MET, metabolic equivalent task. *Adjusted for age, sex, smoking status, alcohol status, meat and fish intake, fruit and vegetable intake, low‐density lipoprotein cholesterol, high‐density lipoprotein, and diabetes (diagnosed by doctor OR using insulin medication OR glucose ≥7.0 mmol/L OR hemoglobin A1C ≥ 48 mmol/mol (6.5%).^†^,^‡^,^§^Odds ratio, 95% CI, and *P* value for odds ratio, are provided for difference in physical activity status for each category of genetic liability on the odds of hypertension. The values are derived from logistic regression models.

### Sensitivity Analysis Using Accelerometer Data

The results of this sensitivity analysis are provided in the supplemental material section (Tables S7‐S9).

## DISCUSSION

The main findings in this study are that (1) 4 favorable adiposity genetic variants are significantly associated with physical activity level; (2) favorable adiposity genetic liability protects against stage 2 hypertension depending on the individual's BMI; and (3) in individuals with high BMI, physical activity provides additional protection against stage 2 hypertension independent of genetic liability. Our study provided insight into the complex relationship between favorable adiposity, BMI, physical activity, and the development of hypertension.

We identified 4 SNPs associated with physical activity none of which were implicated in physical activity previously. The exact mechanism by which these SNPs potentially enhance physical activity remains to be elucidated. However, for 1 SNP (rs4684847), the nearest gene is peroxisome proliferator‐activated receptor gamma (*PPARG*), which influences muscle metabolism and insulin sensitivity,^43^ which are crucial for physical activity and exercise performance^44^, ^45^ implying a potential path to physical activity for this SNP. Three of these physical activity implicated SNPs (rs987469, rs4684847, rs12369179) are associated with BMI and the same allele that is associated with enhanced physical activity is also associated with a lower BMI, implying that these SNPs may increase BMI by reducing physical activity levels. Future research focusing on the relationship between physical activity, BMI, and these SNPs could shed more light on the matter. These SNPs were excluded from our analyses as among high body mass subgroups we observed an association between genetic liability and physical activity that was attenuated after excluding these SNPs.

In the current study we replicated previous findings that favorable adiposity genetic liability protects against hypertension^13^, ^14^ and we additionally found that the combined effect of physical activity and high genetic liability, predominantly in individuals with high BMI, enhances the protection against hypertension. High BMI groups of individuals carry up to 13% less risk of hypertension when carrying optimal status of both genetic and physical activity compared with when both genetic and physical activity are suboptimal. A person with high BMI could reduce risk of hypertension by 5% to 7% by engaging in physical activity alone when genetic liability status is kept constant. In our previous work^11^ focusing on adiposity genetic liability, we observed a 10% reduction in risk of hypertension as a result of physical activity. However, our previous work did not differentiate the high and low body mass groups. The 5% to 7% risk reduction we observed as a result of physical activity in the current study is personalized depending on BMI and genetic liability status. This reduction of risk of hypertension is not observed in the low body mass group. Our previous work also demonstrated that a combination of suboptimal obesity genetic liability and suboptimal physical activity enhances risk of hypertension.^11^ However, our previous work did not differentiate between specific obesity subgroups, and it was focused on obesity genetic liability rather than favorable adiposity genetic liability (one is known to increase risk of hypertension and the other reduces risk of hypertension).^11^ The current research additionally examines how favorable adiposity genetic liability reduces hypertension stratified by BMI and physical activity.

Our study is a step forward in personalized medicine compared with previous community intervention programs such as the “Pas‐a‐Pas” community intervention that observed a 6.63 mm Hg reduction in SBP^46^ as a result of physical activity. Reduction in hypertension risk could alleviate the burden on the National Health Service, by decreasing hospital admissions, the need for antihypertensive medications,^47^ and the incidence of hypertension‐related diseases such as stroke.^48^ Obesity, a major risk factor for hypertension, creates a substantial burden to the National Health Service, with obesity‐related conditions costing the National Health Service £6.5 billion annually.^49^ Our study shows that physical activity interventions would be beneficial in reducing risk of hypertension only for high body mass groups, suggesting that physical activity programs that aim to reduce hypertension should target high body mass groups essentially as there is no benefit for low body mass groups. Our study paves the way for precision medicine and improved classification of individuals at risk. The findings in our study provide potential impact on better management and prevention of hypertension among high body mass individuals. The results could guide health professionals to encourage high body mass individuals to engage in physical activity. By targeting high body mass groups, physical activity programs could mitigate the costs, improve patient outcomes, and enhance public health. Clinicians could recommend that obese individuals should aim for either ≥150 METs of moderate activity (eg, 37.5 hours of brisk walking per week) or ≥75 METs of vigorous activity (eg, ~10 hours of running per week) to reduce hypertension risk (Table 5).

The protective effect of genetic liability on hypertension does not exist among individuals with low BMI no matter what their physical activity level is. One potential explanation for this could be that BMI reflects both muscle mass and fat mass.^50^ It is possible that the individuals we stratified in the low BMI group could have low muscle mass as well as low fat mass. Low muscle mass could be an indicator of subclinical underlying issues with potential impact on hypertension (eg, sarcopenia^51^ or chronic obstructive pulmonary disease^52^, ^53^, ^54^). We have adjusted for factors such as smoking (strong risk factors for chronic obstructive pulmonary disease) as well as age and diet to mitigate the effect of such confounders; however, unknown confounding effects might still exist.

The main strengths of this study include the UKB's large sample size and rich phenotyping, which provide optimal statistical power to investigate the relationship between genetic factors and complex outcomes. Another strength is the number of favorable adiposity variants used in this study using the most recent insight into capturing more accurate estimation of favorable adiposity genetic liability. Another strength is in the use of K‐means algorithm ensuring that the self‐reported ancestry data matches with the genetically derived ancestry and thus providing improved accuracy into the use of the European ancestry sample of the UKB. A key limitation of the study is that the UKB cohort is not nationally representative and is generally healthier than the overall UK population,^55^ which can introduce a ‘healthy volunteer’ bias in all associations.^56^ An additional limitation of this study is that the sample represents a healthier subset of the UKB cohort, which may further restrict the generalizability of our findings to the broader, more diverse population.

Although our findings are promising, further research is needed to explore the underlying mechanisms. Future studies should also investigate whether similar patterns are observed in different populations and age groups and how these findings could be translated into practical interventions for hypertension prevention.

## CONCLUSIONS

The findings from this study demonstrate that the protective effect of favorable adiposity varies depending on the body mass and physical activity status. The finding underscores the significant role of physical activity in mitigating hypertension risk, particularly in individuals with high body mass and favorable adiposity genetic liability. Further research using ancestry‐specific genetic variants could help understanding how these results apply to other ethnic populations.

## Sources of Funding

UKB genotyping was supported by the British Heart Foundation (grant SP/13/2/30111) for large‐scale comprehensive genotyping of UKB for cardiometabolic traits and diseases: UK CardioMetabolic Consortium.

## Disclosures

None.

## Supporting information

Supplemental Methods
Tables S1–S9
Figure S1
