# Supplementary material for: Physical Activity and Favorable Adiposity Genetic Liability Reduce the Risk of Hypertension Among High Body Mass Individuals
Source: J Am Heart Assoc. 2025 Oct 23;14(21):e040701. doi: 10.1161/JAHA.124.040701 (PMC12684782; doi:10.1161/JAHA.124.040701)
Supplement: Supplementary file 1 — Supplemental Methods Tables S1–S9 Figure S1 [file JAH3-14-e040701-s001.pdf]

Supplemental Materials

Supplementary Methods

Accelerometer data collection

Accelerometer data was measured centrally by the UKB between 2013 and 2015 using the Axivity AX3 wrist-worn triaxial accelerometer (Axivity Ltd., Newcastle, UK) [39]. This was assessed years after the first assessment of the UKB participants. Participants were requested to wear the monitor continuously for seven consecutive days. The sensor captured acceleration for seven days at a frequency of 100Hz with a dynamic range of ±8g, and the signals were calibrated to gravity. We filtered out participants who did not wear the device sufficiently long (*f.90015*) to get a stable measure of their accelerometer-registered activity (n= 6995) and whose data could not be calibrated (*f.90016*; n= 11). We used the overall acceleration average since it measured the total time spent across all levels of physical activity intensity [39]. Following the application of our exclusion criteria described in the methods section of our manuscript (**Figure S1**), we were left with a final sample size of 46,051 participants. For the purpose of this sensitivity analysis, we assumed that the accelerometer data collected from 2013-2015 was an accurate proxy for the physical activity of the participants assessed back in time during the first UKB assessment (2006-2010) where the blood pressure data was collected. We categorised the overall acceleration within the population distribution based on their median values into physically active (27.61 - 204.07 milli-gravity) and physically inactive (3.99 - 27.60 milli-gravity).

We examined the combined effects of genetic liability and accelerometer-registered activity on the odds of hypertension stratified by (1) body mass, and (2) age, sex and body mass index. We also tested the pure effect of accelerometer-registered activity on the observed effects by comparing the modulatory effect of accelerometer-

registered activity on the association of genetic liability and hypertension within subgroups with similar BMI and genetic liability.

Supplementary Results and Discussion

Stratified by BMI, we performed sensitivity analysis to replicate physical activity analysis using accelerometer data instead of self-reported. We observed lower odd of hypertension among high body mass participants who had high accelerometer-registered activity accompanied by moderate or high genetic liability as compared with participants with low accelerometer-registered activity and low genetic liability (Adjusted $OR_{\text{moderate genetic liability, physically active}}$ = 0.88; 95% CI= 0.80, 0.96; *P*=3.63 $x10^{-3}$; Adjusted $OR_{\text{high genetic liability, physically active}}$ = 0.80; 95% CI= 0.73, 0.88; *P*=1.60 $x10^{-6}$). Similar results were also observed among participants with low body mass (Adjusted $OR_{\text{high genetic liability, physically active}}$ = 0.82; 95% CI= 0.73, 0.91; *P*=3.42 $x10^{-4}$; **Table S7**).

This analysis shows a 12-20% less odds of hypertension depending on the genetic status and accelerometer-registered activity. This is comparable to our findings of 7-13% less odds of hypertension depending on the genetic status and self-reported physical activity. However, with the self-reported data, we did not observe any statistically significant changes in the low body mass individuals. This is only seen using accelerometer data. While accelerometer data provide a more precise estimate of individual activities than self-reported, it should be noted that firstly, the accelerometer data was registered in 2013-2015 while hypertension data was collected in 2006-2010 several years before accelerometer data collection occurred. This creates a challenge to generalize the data obtained from future physical activity in association with baseline hypertension as it poses a risk of bias due to reverse causation. Secondly, the participants were aware that their physical activity being registered and multiple factors such as psychological factors could have played a

role in their activity while wearing the device. In addition, the wide chronological gap between baseline hypertension status and accelerometer assessment could mean change in the health and lifestyle status of the participants from the baseline visit that could have affected the ability to use of the device.

When we investigated individuals within the same categories of BMI and genetic liability status, we observed 10-20% less odds of hypertension across most subgroups who had high accelerometer-registered activity compared with participants with low accelerometer-registered activity **(Table S7**).

This range exceeds the 5-7% reduction seen using self-reported physical activity data. We found that combinations of favourable adiposity genetic liability and optimal accelerometer-registered activity shows association with lower risk of hypertension even for individuals with low body mass. Compared to our findings using the self-reported physical activity, the differences may be attributed to the more accurate and comprehensive measurement of physical activity using accelerometer data [40].

We repeated the analyses above additionally stratified by age and sex (**Table S8)** and observed that among high body mass participants; the odds of hypertension were less among men under the age of 60 for most of the BMI-genetic liability-activity subgroups compared with participants who had low accelerometer-registered activity combined with low genetic liability (**Table S8**). In addition, the odds of hypertension was less among women under the age of 60 years who had high accelerometer-registered activity combined with a high or moderate genetic liability (**Table S8**). No association was observed between any combination of genetic liability and accelerometer-registered activity with the odds of hypertension in men and women older than 60 years (**Table S8).**

Among low body mass participants**,** we observed 27-28% less odds of hypertension among men older than 60 years who had high accelerometer-registered activity with high or low genetic liability. This was made in comparison with our reference group comprised of low body mass men who had low accelerometer-registered activity combined with low genetic liability. Additionally, we observed 29% less odds of hypertension among women older than 60 years who had high accelerometer-registered activity combined with high genetic liability. This was made in comparison with our reference group comprised of low body mass women who had low accelerometer-registered activity combined with low genetic liability **(Table S8).** The observed differences among age and sex subgroups using accelerometer data align with Cho and colleagues [41], who found that higher levels of self-reported physical activity offer greater benefits to women. This underscores the need for tailored physical activity guidelines based on genetic profiles, sex, and age to reduce hypertension risk effectively. Additionally, we quantified the personalised odds of hypertension in different categories of body mass, genetic liability and accelerometer-registered activity (**Table S9**).

Using UK Biobank (UKB) accelerometer data in our study presents several limitations. Firstly, the accelerometer data were not collected at baseline [39], meaning that conditions such as hypertension and other health metrics were measured before collecting physical activity data. This temporal discrepancy could introduce biases, as changes in health status influence physical activity levels. Additionally, participants were aware that their physical activity was being monitored, which could have led to an increase in their activity levels due to the Hawthorne effect [42] that is bias due to awareness of being monitored which can influence patient or student behaviour. Furthermore, the accelerometer data were collected over a short period

(typically seven days), which may not accurately represent long-term physical activity patterns.

Table S1. List of SNPs used for the generation of favourable adiposity genetic liability following pruning.

FAVOURABLE ADIPOSITY					
SNP	Effect Allele	Effect Estimate	SNP	Effect Allele	Effect Estimate
rs12369179	C	0.03	rs12681990	T	0.01
rs4684847	T	0.03	rs6977416	G	0.01
rs62271373	T	0.02	rs4976033	A	0.01
rs72959041	G	0.02	rs12441543	A	0.01
rs12130231	A	0.02	rs12940684	C	0.01
rs7133378	A	0.02	rs113222038	C	0.01
rs4821764	G	0.02	rs11045172	C	0.01
rs13389219	T	0.02	rs2802774	A	0.01
rs2943653	C	0.02	rs7233512	G	0.01
rs7258937	T	0.02	rs9764678	C	0.01
rs72697297	T	0.02	rs10876529	C	0.01
rs972283	A	0.01	rs11664106	T	0.01
rs11135038	G	0.01	rs998584	C	0.01
rs9851766	A	0.01	rs6029180	G	0.01
rs142186653	C	0.01	rs13132853	A	0.01
rs987469	C	0.01	rs573454216	A	0.01
rs2980888	C	0.01	rs4450871	G	0.01
rs30351	G	0.01			

All known SNPs from previously reported genetic variants for favourable adiposity and their effect size have been included [12]. SNPs are pairwise independent and not in linkage disequilibrium. SNP: Single Nucleotide Polymorphism.

Table S2. The relationship between the UK Biobank self-reported ancestry with genetic based clusters following K-means clustering analysis (N=488,247).

**Genetically Derived Clusters**	**UKB Self-Reported Ancestry**						
	**African**	**Asian**	**Chinese**	**missing**	**mixed**	**Other**	**White**
**1**	85	8750	1	253	385	583	71
**2**	11	19	3	357	259	66	131,138
**3**	191	275	1	416	469	1476	96,629
**4**	31	48	1	751	794	528	180,077
**5**	7	11	1	225	190	250	52,166
**6**	7316	1	0	228	554	800	5
**7**	4	370	1497	78	192	653	31

Table S3. Effect of favourable adiposity SNPs and genetic liability on the total amount of MET/minute physical activity

Favourable Adiposity SNPS (**Coded allele**)	Whole Population (N= 210,290)			High body mass Population (N= 132,278)			Low body mass Population (N= 78,012)		
	Effect estimate (β)	95% CI	P- value	Effect estimate (β)	95% CI	P- value	Effect estimate (β)	95% CI	P- value
rs12369179 Ω (C)									
Unadjusted Odds Ratios	-10.13	-21.11 – 0.84	0.07	-14.26	-28.23 – -0.30	0.04	-0.17	-17.85 – 17.52	0.99
Adjusted Odds Ratios†	-10.63	-21.57 – 0.32	0.06	-14.90	-28.81 – -0.99	0.04	0.13	-17.52 – 17.78	0.99
rs4684847§ (C)									
Unadjusted Odds Ratios	16.36	5.29 – 27.43	3.77x10^{-3}	18.92	4.98 – 32.86	7.81x10^{-3}	13.24	-4.91 – 31.39	0.15
Adjusted Odds Ratios†	15.89	4.85 – 26.93	4.79x10^{-3}	18.77	4.88 – 32.65	8.06x10^{-3}	12.66	-5.46 – 30.77	0.17
rs62271373 (T)									
Unadjusted Odds Ratios	-5.15	-16.17 – 5.88	0.36	-8.31	-22.21 – 5.59	0.24	0.50	-17.56 – 18.57	0.96
Adjusted Odds Ratios†	-5.85	-16.85 – 5.14	0.30	-8.81	-22.65 – 5.03	0.21	-0.35	-18.37 – 17.67	0.97
rs72959041 (G)									
Unadjusted Odds Ratios	-1.75	-12.71 – 9.22	0.76	-1.11	-15.00 – 12.79	0.88	-1.42	-19.23 – 16.40	0.88
Adjusted Odds Ratios†	-1.34	-12.28 – 9.60	0.81	-0.57	-14.40 – 13.27	0.94	-0.86	-18.64 – 16.91	0.92
rs12130231 (A)									
Unadjusted Odds Ratios	-9.69	-20.95 – 1.57	0.09	-13.44	-27.65 – 0.77	0.06	-1.49	-19.92 – 16.93	0.87
Adjusted Odds Ratios†	-10.17	-21.40 – 1.06	0.08	-13.61	-27.76 – 0.54	0.06	-2.29	-20.67 – 16.10	0.81
rs7133378 (G)									
Unadjusted Odds Ratios	5.60	-5.65 – 16.84	0.33	-0.71	-14.86 – 13.45	0.92	17.63	-0.84 – 36.09	0.06
Adjusted Odds Ratios†	5.19	-6.03 – 16.41	0.37	-0.14	-14.24 – 13.96	0.98	16.44	-1.99 – 34.86	0.08
rs4821764 (G)									
Unadjusted Odds Ratios	-2.52	-13.72 – 8.69	0.66	-7.59	-21.71 – 6.52	0.29	7.68	-10.69 – 26.04	0.41
Adjusted Odds Ratios†	-2.31	-13.49 – 8.86	0.69	-7.17	-21.23 – 6.88	0.32	8.26	-10.07 – 26.58	0.38
rs13389219 (C)									
Unadjusted Odds Ratios	3.83	-7.44 – 15.09	0.51	1.90	-12.31 – 16.12	0.79	7.36	-11.08 – 25.79	0.43
Adjusted Odds Ratios†	3.50	-7.74 – 14.73	0.54	2.52	-11.64 – 16.67	0.73	6.25	-12.15 – 24.65	0.51
rs2943653 (C)									
Unadjusted Odds Ratios	2.61	-8.60 – 13.81	0.65	4.09	-10.06 – 18.24	0.57	-0.07	-18.38 – 18.23	0.99
Adjusted Odds Ratios†	1.88	-9.29 – 13.06	0.74	2.90	-11.19 – 16.99	0.69	-0.18	-18.44 – 18.08	0.99
rs7258937 (C)									
Unadjusted Odds Ratios	-0.82	-12.02 – 10.39	0.89	-0.85	-15.00 – 13.29	0.91	0.81	-17.50 – 19.13	0.93
Adjusted Odds Ratios†	-1.45	-12.62 – 9.73	0.80	-1.17	-15.26 – 12.91	0.87	0.04	-18.24 – 18.32	1.00
rs72697297 (T)									
Unadjusted Odds Ratios	-1.40	-12.47 – 9.67	0.80	-3.80	-17.76 – 10.16	0.59	2.46	-15.68 – 20.59	0.79
Adjusted Odds Ratios†	-1.43	-12.47 – 9.62	0.80	-2.73	-16.63 – 11.17	0.70	0.99	-17.11 – 19.09	0.92
rs972283 (A)									
Unadjusted Odds Ratios	-0.76	-11.99 – 10.48	0.90	-0.03	-14.20 – 14.14	1.00	0.85	-17.56 – 19.25	0.93
Adjusted Odds Ratios†	-1.25	-12.46 – 9.96	0.83	-0.60	-14.71 – 13.514	0.93	1.11	-17.25 – 19.48	0.91
rs11135038 (T)									
Unadjusted Odds Ratios	-7.41	-18.55 – 3.73	0.19	-9.74	-23.79 – 4.30	0.17	-2.23	-20.48 – 16.02	0.81
Adjusted Odds Ratios†	-7.97	-19.08 – 3.14	0.16	-9.74	-23.73 – 4.25	0.17	-2.92	-21.13 – 15.29	0.75
rs9851766 (A)									
Unadjusted Odds Ratios	-5.18	-16.27 – 5.91	0.36	-5.48	-19.53 – 8.57	0.45	-1.55	-19.57 – 16.47	0.87
Adjusted Odds Ratios†	-5.45	-16.51 – 5.61	0.33	-5.39	-19.38 – 8.60	0.45	-1.55	-19.53 – 16.43	0.87
rs142186653 (A)									
Unadjusted Odds Ratios	-1.68	-12.98 – 9.62	0.77	-0.20	-14.43 – 14.02	0.98	-2.73	-21.28 – 15.81	0.77
Adjusted Odds Ratios†	-2.48	-13.75 – 8.79	0.67	-0.89	-15.05 – 13.28	0.90	-3.35	-21.86 – 15.15	0.72

Effect estimates (β) and 95% confidence interval (CI) for favourable adiposity SNPs and favourable adiposity genetic liability are estimates from linear regression models. † Adjusted for age and sex. § Favourable adiposity SNPs associated with increased physical activity from the linear regression model. Ω Favourable adiposity SNPs associated with reduced physical activity from the linear regression model.

Table S3. Effect of favourable adiposity SNPs and genetic liability on the total amount of MET/minute physical activity (continued)

Favourable Adiposity SNPS	Whole Population (N= 210,290)			High body mass Population (N= 132,278)			Low body mass Population (N= 78,012)		
	Effect estimate (β)	95% CI	P- value	Effect estimate (β)	95% CI	P- value	Effect estimate (β)	95% CI	P- value
rs987469 $^{\Omega}$ (C)									
Unadjusted Odds Ratios	-12.01	-23.22 – -0.81	0.04	-17.45	-31.60 – -3.29	0.02	-2.16	-20.44 – 16.12	0.82
Adjusted Odds Ratios†	-12.04	-23.22 – -0.87	0.03	-18.01	-32.10 – -3.91	0.01	-1.27	-19.51 – 16.98	0.89
rs2980888 (T)									
Unadjusted Odds Ratios	-5.50	-16.70 – 5.71	0.34	-6.04	-20.21 – 8.13	0.40	-0.38	-18.62 – 17.86	0.97
Adjusted Odds Ratios†	-6.62	-17.79 – 4.55	0.25	-6.73	-20.84 – 7.39	0.35	-1.19	-19.39 – 17.01	0.90
rs30351 (G)									
Unadjusted Odds Ratios	7.43	-4.14 – 19.00	0.21	8.43	-6.16 – 23.01	0.26	5.98	-12.96 – 24.93	0.54
Adjusted Odds Ratios†	6.50	-5.04 –18.04	0.27	7.09	-7.44 – 21.61	0.34	4.95	-13.96 – 23.85	0.61
rs12681990 (T)									
Unadjusted Odds Ratios	-4.36	-15.89 – 7.17	0.46	-13.68	-28.24 – 0.89	0.07	12.01	-6.82 – 30.84	0.21
Adjusted Odds Ratios†	-4.88	-16.38 – 6.62	0.41	-13.80	-28.31 – 0.70	0.06	11.17	-7.62 – 29.96	0.24
rs6977416 (G)									
Unadjusted Odds Ratios	2.65	-8.69 – 14.00	0.65	-0.74	-15.05 – 13.57	0.92	8.36	-10.20 – 26.92	0.38
Adjusted Odds Ratios†	2.63	-8.69 – 13.94	0.65	-1.10	-15.35 – 13.15	0.88	8.49	-10.03 – 27.01	0.37
rs4976033 (A)									
Unadjusted Odds Ratios	-6.59	-17.80 – 4.62	0.25	-13.46	-27.59 – 0.66	0.06	6.34	-12.04 – 24.72	0.50
Adjusted Odds Ratios†	-6.69	-17.87 – 4.49	0.24	-13.77	-27.84 – 0.29	0.06	6.85	-11.49 – 25.19	0.46
rs12441543 (G)									
Unadjusted Odds Ratios	0.87	-10.39 – 12.13	0.88	2.24	-11.95 – 16.44	0.76	-0.36	-18.82 – 18.09	0.97
Adjusted Odds Ratios†	0.24	-10.99 – 11.47	0.97	1.36	-12.77 – 15.49	0.85	-0.78	-19.19 – 17.64	0.93
rs12940684 (C)									
Unadjusted Odds Ratios	5.08	-6.22 – 16.39	0.38	0.65	-13.58 – 14.89	0.93	14.50	-4.07 – 33.07	0.13
Adjusted Odds Ratios†	4.48	-6.80 – 15.76	0.44	0.03	-14.15 – 14.21	1.00	14.10	-4.43 – 32.63	0.14
rs113222038 (C)									
Unadjusted Odds Ratios	-8.02	-19.15 – 3.11	0.16	-7.06	-21.11 – 6.99	0.33	-9.77	-27.94 – 8.40	0.29
Adjusted Odds Ratios†	-7.40	-18.49 – 3.70	0.19	-6.27	-20.27 – 7.72	0.38	-9.17	-27.30 – 8.96	0.32
rs11045172 (A)									
Unadjusted Odds Ratios	3.66	-7.41 – 14.74	0.52	1.34	-12.60 – 15.28	0.85	9.09	-9.09 – 27.27	0.33
Adjusted Odds Ratios†	3.68	-7.36 – 14.73	0.51	1.80	-12.08 – 15.69	0.80	8.85	-9.29 – 26.99	0.34
rs2802774 (C)									
Unadjusted Odds Ratios	-1.83	-12.97 – 9.32	0.75	-1.78	-15.83 – 12.28	0.80	0.08	-18.17 – 18.33	0.99
Adjusted Odds Ratios†	-2.00	-13.11 – 9.12	0.72	-2.38	-16.38 – 11.61	0.74	0.87	-17.33 – 19.08	0.93
rs7233512 (G)									
Unadjusted Odds Ratios	3.74	-7.53 – 15.00	0.52	2.39	-11.83 – 16.61	0.74	8.31	-10.10 – 26.72	0.38
Adjusted Odds Ratios†	3.34	-7.89 – 14.58	0.56	2.47	-11.69 – 16.63	0.73	7.78	-10.59 – 26.15	0.41
rs9764678 (T)									
Unadjusted Odds Ratios	-0.52	-11.72 – 10.68	0.92	6.41	-7.69 – 20.51	0.37	-12.14	-30.54 – 6.25	0.20
Adjusted Odds Ratios†	0.07	-11.11 – 11.24	0.99	6.93	-7.12 – 20.97	0.33	-11.29	-29.65 – 7.06	0.23
rs10876529 (T)									
Unadjusted Odds Ratios	-6.98	-18.13 – 4.17	0.22	-2.83	-16.89 – 11.23	0.69	-13.60	-31.87 – 4.68	0.15
Adjusted Odds Ratios†	-6.70	-17.82 – 4.43	0.24	-1.99	-15.98 – 12.01	0.78	-13.22	-31.45 – 5.01	0.16
rs11664106 (A)									
Unadjusted Odds Ratios	0.30	-10.84 – 11.44	0.96	2.23	-11.81 – 16.28	0.76	-1.04	-19.29 – 17.20	0.91
Adjusted Odds Ratios†	0.15	-10.96 – 11.26	0.98	2.77	-11.22 – 16.76	0.70	-1.33	-19.53 – 16.88	0.89

Effect estimates (β) and 95% confidence interval (CI) for favourable adiposity SNPs and favourable adiposity genetic liability are estimates from linear regression models. † Adjusted for age and sex. § Favourable adiposity SNPs associated with increased physical activity from the linear regression model. $^{\Omega}$ Favourable adiposity SNPs associated with reduced physical activity from the linear regression model.

Table S3. Effect of favourable adiposity SNPs and genetic liability on the total amount of MET/minute physical activity (continued)

Favourable Adiposity SNPS	Whole Population (N= 210,290)			High body mass Population (N= 132,278)			Low body mass Population (N= 78,012)		
	Effect estimate (β)	95% CI	P- value	Effect estimate (β)	95% CI	P- value	Effect estimate (β)	95% CI	P- value
rs998584 (C)									
Unadjusted Odds Ratios	-2.05	-13.30 – 9.19	0.72	0.77	-13.41 –14.95	0.92	-4.84	-23.25 – 13.57	0.61
Adjusted Odds Ratios†	-3.10	-14.31 – 8.12	0.59	-0.29	-14.41 – 13.83	0.97	-5.80	-24.17 – 12.57	0.54
rs6029180 (A)									
Unadjusted Odds Ratios	10.18	-0.95 – 21.31	0.07	7.67	-6.33 – 21.67	0.28	17.66	-0.65 – 35.97	0.06
Adjusted Odds Ratios†	10.15	-0.96 – 21.25	0.07	8.08	-5.86 – 22.02	0.26	17.79	-0.48 – 36.06	0.06
rs13132853 (A)									
Unadjusted Odds Ratios	4.54	-6.63 – 15.70	0.43	-1.41	-15.50 – 12.68	0.84	15.24	-3.01 – 33.50	0.10
Adjusted Odds Ratios†	4.82	-6.31 – 15.96	0.40	-1.55	-15.58 – 12.48	0.83	16.10	-2.12 – 34.32	0.08
rs573454216 Ω (G)									
Unadjusted Odds Ratios	-10.63	-21.87 – 0.61	0.06	-15.53	-29.70 – -1.36	0.03	-1.95	-20.34 – 16.45	0.84
Adjusted Odds Ratios†	-10.63	-21.84 – 0.58	0.06	-15.99	-30.11 – -1.88	0.03	-1.31	-19.67 – 17.05	0.89
rs4450871 (A)									
Unadjusted Odds Ratios	-3.47	-14.73 – 7.78	0.55	-6.61	-20.81 – 7.59	0.36	2.50	-15.90 – 20.90	0.79
Adjusted Odds Ratios†	-3.38	-14.61 – 7.84	0.56	-6.91	-21.05 – 7.24	0.34	3.49	-14.88 – 21.85	0.71
Favourable Adiposity genetic liability									
Unadjusted Odds Ratios	-6.07	-17.39 – 5.26	0.29	-15.39	-29.67 – -1.11	0.03	17.51	-1.04 – 36.07	0.06
Adjusted Odds Ratios†	-7.82	-19.12 – 3.47	0.18	-16.18	-30.40 – -1.96	0.03	16.34	-2.17 – 34.86	0.08
Favourable Adiposity genetic liability δ									
Unadjusted Odds Ratios	-5.29	-16.72 – 6.15	0.37	-13.71	-28.12 – 0.71	0.06	16.19	-2.56 – 34.95	0.09
Adjusted Odds Ratios†	-6.96	-18.36 – 4.45	0.23	-14.21	-28.57 – 0.15	0.05	14.74	-3.97 – 33.45	0.12

Effect estimates (β) and 95% confidence interval (CI) for favourable adiposity SNPs and favourable adiposity genetic liability are estimates from linear regression models. † Adjusted for age and sex. § Favourable adiposity SNPs associated with increased physical activity from the linear regression model. Ω Favourable adiposity SNPs associated with reduced physical activity from the linear regression model. δ Favourable adiposity genetic liability estimated without the physical activity-associated SNPs.

Table S4. Association between the physical activity-associated favourable adiposity SNPs and adiposity

Favourable Adiposity SNPS (Coded allele)	Whole Population (N= 210,290)		
	Effect estimate (β)	95% CI	P- value
rs12369179 Ω (C)			
Unadjusted Odds Ratios	0.06	0.04 - 0.08	1.47 $\times 10^{-9}$
Adjusted Odds Ratios†	0.06	0.04 - 0.08	3.80 $\times 10^{-9}$
rs4684847§ (C)			
Unadjusted Odds Ratios	0.04	0.02 - 0.05	3.50 $\times 10^{-4}$
Adjusted Odds Ratios†	0.03	0.02 - 0.05	4.20 $\times 10^{-4}$
rs987469 Ω (C)			
Unadjusted Odds Ratios	0.03	0.01 - 0.05	7.23 $\times 10^{-3}$
Adjusted Odds Ratios†	0.03	0.01 – 0.05	6.22 $\times 10^{-3}$
rs573454216 Ω (G)			
Unadjusted Odds Ratios	0.01	-0.01 - 0.03	0.29
Adjusted Odds Ratios†	0.01	-0.01 - 0.03	0.29

Effect estimates (β) and 95% confidence interval (CI) for physical activity-associated favourable adiposity SNPs are estimates from linear regression models. † Adjusted for age and sex. § Favourable adiposity SNPs associated with increased physical activity from the linear regression model. Ω Favourable adiposity SNPs associated with reduced physical activity from the linear regression model.

Table S5. The effect of genetic liability on hypertension stratified for body mass and physical activity status (excluding physical activity-related SNPs)

**MAIN ANALYSIS (n=210,290)**																									
Genetic Liability	**High body mass AND Low body mass**					**High body mass (n= 132,278)**										**Low body mass (n= 78,012)**									
	Whole Population (n=210,290)					Whole Population (n=132,278)				Physically Active (n= 13,425)			Physically Inactive (n=18,853)			Whole Population (n=78,012)				Physically Active (n=70,448)			Physically Inactive (n=7564)		
	Odds Ratio	95% CI	*P*- Value for Odds Ratio	Interaction *P*-Value*	Interaction *P*-Value §	Odds Ratio	95% CI	*P*- Value for Odds Ratio	Interaction *P*-Value*	Odds Ratio	95% CI	*P*- Value for Odds Ratio	Odds Ratio	95% CI	*P*- Value for Odds Ratio	Odds Ratio	95% CI	*P*- Value for Odds Ratio	Interaction *P*-Value*	Odds Ratio	95% CI	*P*- Value for Odds Ratio	Odds Ratio	95% CI	*P*- Value for Odds Ratio
**Unadjusted Odds Ratios**																									
Favourable Adiposity ‡	0.98	0.97 - 0.99	1.25×10^{-6}	0.27	2.16×10^{-3}	0.97	0.96 - 0.98	4.52×10^{-9}	0.60	0.97	0.95 - 0.98	3.83×10^{-9}	0.98	0.96 - 1.01	0.27	0.97	0.96 - 0.99	3.71×10^{-4}	0.46	0.97	0.96 - 0.99	4.32×10^{-4}	0.98	0.94 - 1.03	0.48
**Minimally Adjusted Odds Ratios [a]**																									
Favourable Adiposity ‡	0.97	0.96 - 0.98	2.80×10^{-10}	0.91	4.20×10^{-3}	0.96	0.95 - 0.97	1.62×10^{-11}	0.83	0.96	0.95 - 0.97	1.30×10^{-10}	0.97	0.94 - 1.00	3.49×10^{-2}	0.97	0.95 - 0.98	1.56×10^{-5}	0.92	0.97	0.95 - 0.98	4.05×10^{-5}	0.97	0.92 - 1.02	0.18
**Adjusted Odds Ratios [b]**																									
Favourable Adiposity ‡	0.98	0.97 - 0.99	1.10×10^{-4}	0.79	8.93×10^{-3}	0.97	0.96 - 0.98	7.94×10^{-8}	0.88	0.97	0.96 - 0.98	2.34×10^{-7}	0.98	0.95 - 1.01	0.11	0.96	0.95 - 0.98	7.37×10^{-6}	0.97	0.97	0.95 - 0.98	3.28×10^{-5}	0.95	0.90 - 1.00	0.07
**SENSITIVITY ANALYSIS: Excluded 24,205 participants on BP lowering medication (n= 186,085)**																									
Genetic Liability	**High body mass AND Low body mass**					**High body mass (n= 113,241)**										**Low body mass (n=67,722)**									
	Whole Population (n=186,085)					Whole Population (n= 113,241)				Physically Active (n=97,515)			Physically Inactive (n=15,726)			Whole Population (n=72,844)				Physically Active (n=65,865)			Physically Inactive (n= 6979)		
**Unadjusted Odds Ratios**																									
Favourable Adiposity ‡	0.99	0.98 – 1.00	2.85×10^{-3}	0.47	9.70×10^{-4}	0.97	0.96 - 0.99	1.88×10^{-5}	0.99	0.97	0.96 - 0.99	3.12×10^{-5}	0.98	0.95 - 1.01	0.28	0.98	0.97 - 1.00	4.78×10^{-2}	0.24	0.98	0.96 - 1.00	3.05×10^{-2}	1.01	0.95 - 1.06	0.79
**Minimally Adjusted Odds Ratios [a]**																									
Favourable Adiposity ‡	0.98	0.97 - 0.99	1.13×10^{-5}	0.91	2.38×10^{-3}	0.97	0.96 - 0.98	3.56×10^{-5}	0.55	0.97	0.96 - 0.98	2.48×10^{-6}	0.97	0.94 - 1.00	0.06	0.98	0.96 - 0.99	5.42×10^{-3}	0.57	0.98	0.96 - 0.99	4.95×10^{-3}	0.99	0.94 - 1.05	0.74
**Adjusted Odds Ratios [b]**																									
Favourable Adiposity ‡	0.99	0.98 – 1.00	1.26×10^{-4}	0.94	4.18×10^{-3}	0.97	0.96 - 0.99	4.16×10^{-5}	0.60	0.97	0.96 - 0.99	1.29×10^{-4}	0.98	0.94 - 1.01	0.16	0.97	0.96 - 0.99	3.02×10^{-3}	0.66	0.97	0.96 - 0.99	3.80×10^{-3}	0.98	0.92 - 1.03	0.42

‡ Estimated genetic liability without favourable adiposity SNPs associated with physical activity (rs12369179; rs4684847; rs987469; rs573454216). Odds ratios were presented for the effect of

physical activity on the genetic liability of favourable adiposity on hypertension according to obesity status. Two groups of physical activity were considered (physically active vs physically inactive).

a Adjusted for age and sex. b Adjusted for age, sex, smoking status, alcohol status, meat and fish intake, fruit and vegetable intake, low-density lipoprotein cholesterol, high-density lipoprotein, and diabetes (diagnosed by doctor OR using insulin medication OR glucose ≥ 7.0mmol/l OR HbA1C ≥ 48mmol/mol (6.5%).

Physical Activity was defined as METs for moderate activity ≥ 150 OR METs for vigorous activity ≥ 75 OR summed METs for all activity ≥ 600

Table S6. Joint effects of genetic liability and physical activity on the odds of hypertension stratified by the body mass index (Estimated genetic liability using all favourable adiposity SNPs)

Obesity Status	FA Genetic Liability Categories	Physical Activity Status	Hypertensive (n)	Non-Hypertensive (n)	% Hypertensive	Unadjusted			Minimally Adjusted [a]			Adjusted [b]		
						Odds Ratio [c]	95% CI [d]	*P*-value for Odds Ratio*	Odds Ratio [c]	95% CI [d]	*P*-value for Odds Ratio*	Odds Ratio [c]	95% CI [d]	*P*-value for Odds Ratio*
High body mass (n=132,278)	Low	Physically Inactive	3272	2760	54.24	1 (reference)			1 (reference)			1 (reference		
	Low	Physically Active	20,116	17,012	54.18	1.00	0.94 - 1.05	0.93	0.92	0.87 - 0.98	5.57 x10^{-3}	0.94	0.89 - 1.00	4.24 x10^{-2}
	Moderate	Physically Inactive	3334	2872	53.72	0.98	0.91 - 1.05	0.56	0.97	0.90 - 1.05	0.48	0.99	0.92 - 1.06	0.74
	Moderate	Physically Active	20,037	17,780	52.98	0.95	0.90 - 1.00	6.86 x10^{-2}	0.88	0.83 - 0.93	3.94 x10^{-6}	0.90	0.85 - 0.95	3.69 x10^{-4}
	High	Physically Inactive	3488	3127	52.73	0.94	0.88 - 1.01	8.79 x10^{-2}	0.91	0.84 - 0.97	7.93 x10^{-3}	0.92	0.86 - 0.99	3.44 x10^{-2}
	High	Physically Active	20,115	18,365	52.27	0.92	0.88 - 0.98	4.39 x10^{-3}	0.84	0.80 - 0.89	4.25 x10^{-9}	0.88	0.83 - 0.93	6.63 x10^{-6}
Low body mass (n=78,012)	Low	Physically Inactive	856	1809	32.12	1 (reference)			1 (reference)			1 (reference		
	Low	Physically Active	8435	15,846	34.74	1.13	1.03 - 1.23	6.95 x10^{-3}	1.04	0.95 - 1.14	0.35	1.05	0.96 - 1.15	0.29
	Moderate	Physically Inactive	796	1691	32.01	1.00	0.89 - 1.12	0.93	0.99	0.87 - 1.12	0.81	0.97	0.86 - 1.10	0.68
	Moderate	Physically Active	7957	15,628	33.74	1.08	0.99 - 1.17	9.38 x10^{-2}	1.00	0.91 - 1.09	0.91	1.00	0.91 - 1.09	0.97
	High	Physically Inactive	784	1628	32.50	1.02	0.91 - 1.15	0.77	0.97	0.86 - 1.10	0.68	0.97	0.85 - 1.09	0.57
	High	Physically Active	7518	15,064	33.29	1.06	0.97 - 1.15	0.22	0.97	0.88 - 1.06	0.47	0.97	0.88 - 1.06	0.48

Odds ratios show the risk of prevalent stage 2 hypertension for participants belonging to each combination compared with the reference group (Low genetic liability combined with physically Inactive).

Abbreviations: FA, Favourable Adiposity.

a Adjusted for age and sex.

b Adjusted for age, sex, smoking status, alcohol status, meat and fish intake, fruit and vegetable intake, low-density lipoprotein cholesterol, high-density lipoprotein, and diabetes (diagnosed by doctor OR using insulin medication OR glucose ≥ 7.0mmol/l OR HbA1C ≥ 48mmol/mol (6.5%).

c , d, *, Odds ratio, 95% confidence interval and p-value for odds ratio, are provided for the joint association between favourable adiposity genetic liability and physical activity status on the risk of hypertension. The values are derived from logistic regression models.

Physical Activity was defined as METs for moderate activity ≥ 150 OR METs for vigorous activity ≥ 75 OR summed METs for all activity ≥ 600

Table S7: Sensitivity analysis using accelerometer data

Obesity Status	FA Genetic Liability ‡ Categories	Accelerometer registered activity	Hypertensive (n)	Non-Hypertensive (n)	% Hypertensive	Joint effects of genetic liability and accelerometer-registered activity on hypertension			Impact of accelerometer-measured activity on hypertension by genetic liability		
						Adjusted [a]			Adjusted [a]		
						Odds Ratio [b]	95% CI [c]	*P*-value for Odds Ratio*	Odds Ratio [d]	95% CI [e]	*P*-value [#]
High body mass (n= 26,416)	Low	Physically Inactive	2648	2211	54.50	**1 (reference)**			**1 (reference)**		
	Low	Physically Active	1763	1969	47.24	0.92	0.84 - 1.00	5.87 $\times 10^{-2}$	0.90	0.82 - 0.99	2.53 $\times 10^{-2}$
	Moderate	Physically Inactive	2746`	2276	54.68	0.99	0.92 - 1.08	0.89	**1 (reference)**		
	Moderate	Physically Active	1776	2121	45.57	0.88	0.80 - 0.96	3.63 $\times 10^{-3}$	0.87	0.80 - 0.95	2.83 $\times 10^{-3}$
	High	Physically Inactive	2819	2364	54.39	1.00	0.93 - 1.09	0.96	**1 (reference)**		
	High	Physically Active	1631	2092	43.81	0.80	0.73 - 0.88	1.60 $\times 10^{-6}$	0.80	0.74 - 0.88	2.45 $\times 10^{-6}$
Low body mass (n= 19,635)	Low	Physically Inactive	984	1757	35.90	**1 (reference)**			**1 (reference)**		
	Low	Physically Active	1182	2837	29.41	0.90	0.81 - 1.00	5.34 $\times 10^{-2}$	0.91	0.81 - 1.01	8.03 $\times 10^{-2}$
	Moderate	Physically Inactive	980	1631	37.53	1.04	0.92 - 1.17	0.52	**1 (reference)**		
	Moderate	Physically Active	1112	2708	29.11	0.90	0.80 - 1.00	5.84 $\times 10^{-2}$	0.87	0.78 - 0.97	1.40 $\times 10^{-2}$
	High	Physically Inactive	954	1681	36.20	0.98	0.88 - 1.11	0.79	**1 (reference)**		
	High	Physically Active	1042	2767	27.36	0.82	0.73 - 0.91	3.42 $\times 10^{-4}$	0.82	0.73 - 0.92	6.83 $\times 10^{-4}$

Abbreviations: FA, Favourable Adiposity (‡ Estimated genetic liability without rs12369179, rs4684847, rs987469 and rs573454216).

a Adjusted for age, sex, smoking status, alcohol status, meat and fish intake, fruit and vegetable intake, low-density lipoprotein cholesterol, high-density lipoprotein, and diabetes (diagnosed by doctor OR using insulin medication OR glucose ≥ 7.0mmol/l OR HbA1C ≥ 48mmol/mol (6.5%).

b , c, *, Odds ratio, 95% confidence interval and p-value for odds ratio, are provided for the joint association between favourable adiposity genetic liability and physical activity status on the odds of hypertension compared with the reference group (Low genetic liability combined with physically Inactive). The values are derived from logistic regression models.

d , e, #, Odds ratio, 95% confidence interval and p-value for odds ratio, are provided for difference in accelerometer-registered activity for each category of genetic liability on the odds of hypertension compared with the reference group (comprising of similar genetic liability combined with physically Inactive). The values are derived from logistic regression models.

Physical Activity was derived through median-split overall accelerometer data. The threshold for physically active participants was ≥ 27.61 milli-gravity.

Table S8. Joint effects of genetic liability and accelerometer-registered activity on the odds of hypertension stratified by the age, sex and body mass index

Body Mass	FA Genetic Liability ǂ Categories	Accelerometer-registered activity	Adjusted [a]			Adjusted [a]			Adjusted [a]			Adjusted [a]		
			Men < 60 (n= 8084)			Men ≥ 60 (n= 4952)			Women < 60 (n= 8632)			Women ≥ 60 (n= 4748)		
			Odds Ratio[c]	95% CI [d]	*P*-value for Odds Ratio*	Odds Ratio[c]	95% CI [d]	*P*-value for Odds Ratio*	Odds Ratio[c]	95% CI [d]	*P*-value for Odds Ratio*	Odds Ratio[c]	95% CI [d]	*P*-value for Odds Ratio*
High body mass (n=26,416)	Low	Physically Inactive	**1 (reference)**			**1 (reference)**			**1 (reference)**			**1 (reference)**		
	Low	Physically Active	0.83	0.71 - 0.97	2.07×10^{-2}	1.04	0.83 - 1.31	0.72	0.92	0.79 - 1.08	0.32	0.94	0.76 - 1.18	0.60
	Moderate	Physically Inactive	0.89	0.77 - 1.04	0.14	1.03	0.86 - 1.23	0.76	0.96	0.83 - 1.11	0.57	1.18	0.99 - 1.40	6.68×10^{-2}
	Moderate	Physically Active	0.82	0.70 - 0.96	1.30×10^{-2}	0.99	0.79 - 1.25	0.95	0.84	0.72 - 0.98	3.09×10^{-2}	0.92	0.74 - 1.15	0.45
	High	Physically Inactive	0.85	0.73 - 0.99	3.73×10^{-2}	1.01	0.84 - 1.20	0.94	1.04	0.90 - 1.20	0.59	1.18	0.99 - 1.40	6×10^{-2}
	High	Physically Active	0.76	0.65 - 0.89	7.45×10^{-4}	0.91	0.73 - 1.14	0.42	0.71	0.60 - 0.84	5.17×10^{-5}	0.92	0.74 - 1.16	0.49
Body Mass	FA Genetic Liability ǂ Categories	Physical Activity Status	Men < 60 (n= 4131)			Men ≥ 60 (n=2489)			Women < 60 (n= 8949)			Women ≥ 60 (n= 4066)		
Low body mass (n=19,635)	Low	Physically Inactive	**1 (reference)**			**1 (reference)**			**1 (reference)**			**1 (reference)**		
	Low	Physically Active	0.88	0.69 - 1.12	0.30	0.72	0.54 - 0.94	1.69×10^{-2}	0.99	0.82 - 1.18	0.87	0.93	0.75 - 1.15	0.47
	Moderate	Physically Inactive	1.10	0.85 - 1.43	0.47	0.91	0.70 - 1.19	0.51	1.12	0.91 - 1.38	0.29	0.99	0.79 - 1.23	0.90
	Moderate	Physically Active	0.88	0.69 - 1.13	0.32	0.80	0.60 - 1.06	0.11	0.98	0.82 - 1.18	0.85	0.87	0.70 - 1.08	0.22
	High	Physically Inactive	0.84	0.64 - 1.10	0.19	0.91	0.70 - 1.18	0.48	1.08	0.88 - 1.33	0.47	1.03	0.82 - 1.28	0.83
	High	Physically Active	0.86	0.67 - 1.10	0.22	0.73	0.55 - 0.97	2.95×10^{-2}	0.90	0.75 - 1.09	0.28	0.71	0.57 - 0.88	2.03×10^{-3}

Odds ratios show the odds of prevalent stage 2 hypertension for participants belonging to each combination compared with the reference group (Low genetic liability combined with physically Inactive).

Abbreviations: FA, Favourable Adiposity.

a, Adjusted for age, sex, smoking status, alcohol status, meat and fish intake, fruit and vegetable intake, low-density lipoprotein cholesterol, high-density lipoprotein, and diabetes (diagnosed by doctor OR using insulin medication OR glucose ≥ 7.0mmol/l OR HbA1C ≥ 48mmol/mol (6.5%).

c , d, *, Odds ratio, 95% confidence interval and p-value for odds ratio, are provided for the joint association between favourable adiposity genetic liability and accelerometer-registered activity on the risk of hypertension. The values are derived from logistic regression models.

Physical Activity was derived through median-split overall accelerometer data. The threshold for physically active participants was ≥ *27.61 milli-gravity*.

Table S9: Overview of the combined effect of accelerometer-registered activity and genetic liability as well as pure effect of accelerometer-registered activity on hypertension (Stratified by age, sex and BMI)

Obesity Status	FA Genetic Liability ‡ Categories	Accelerometer-registered activity	Joint effects of genetic liability and accelerometer-registered activity on hypertension			Impact of accelerometer-measured activity on hypertension by genetic liability			Joint effects of genetic liability and accelerometer-registered activity on hypertension			Impact of accelerometer-measured activity on hypertension by genetic liability		
			**Men < 60 years (N=8084)**			**Men < 60 years (N=8084)**			**Women < 60 years (N=8632)**			**Women < 60 years (N=8632)**		
			Adjusted [a]			Adjusted [a]			Adjusted [a]			Adjusted [a]		
			Odds Ratio [b]	95% CI [c]	*P*-value for Odds Ratio*	Odds Ratio [d]	95% CI [e]	*P*-value [#]	Odds Ratio [b]	95% CI [c]	*P*-value for Odds Ratio*	Odds Ratio [d]	95% CI [e]	*P*-value [#]
High body mass (n= 26,416)	Low	Physically Inactive	**1 (reference)**			**1 (reference)**			**1 (reference**)			**1 (reference)**		
	Low	Physically Active	0.83	0.71 - 0.97	2.07×10^{-2}	0.83	0.71 - 0.98	2.44×10^{-2}	0.92	0.79 - 1.08	0.32	0.94	0.80 - 1.10	0.45
	Moderate	Physically Inactive	0.89	0.77 - 1.04	0.14	**1 (reference)**			0.96	0.83 - 1.11	0.57	**1 (reference)**		
	Moderate	Physically Active	0.82	0.70 - 0.96	1.30×10^{-2}	0.92	0.79 - 1.08	0.30	0.84	0.72 - 0.98	3.09×10^{-2}	0.86	0.73 - 1.01	6.16×10^{-2}
	High	Physically Inactive	0.85	0.73 - 0.99	3.73×10^{-2}	**1 (reference)**			1.04	0.90 - 1.20	0.59	**1 (reference)**		
	High	Physically Active	0.76	0.65 - 0.89	7.45×10^{-4}	0.90	0.77 - 1.05	0.19	0.71	0.60 - 0.84	5.17×10^{-5}	0.68	0.58 - 0.79	2.01×10^{-6}
Obesity Status		FA Genetic Liability ‡ Categories	**Men ≥ 60 years (N=2489)**			**Men ≥ 60 years (N=2489)**			**Women ≥ 60 years (N=4066)**			**Women ≥ 60 years (N=4066)**		
			Adjusted [a]			Adjusted [a]			Adjusted [a]			Adjusted [a]		
			Odds Ratio [b]	95% CI [c]	*P*-value for Odds Ratio*	Odds Ratio [d]	95% CI [e]	*P*-value [#]	Odds Ratio [b]	95% CI [c]	*P*-value for Odds Ratio*	Odds Ratio [d]	95% CI [e]	*P*-value [#]
Low body mass (n= 19,635)	Low	Physically Inactive	**1 (reference**)			**1 (reference)**			**1 (reference)**			**1 (reference)**		
	Low	Physically Active	0.72	0.54 - 0.94	1.69×10^{-2}	0.72	0.54 - 0.95	2.21×10^{-2}	0.93	0.75 - 1.15	0.47	0.93	0.75 - 1.15	0.48
	Moderate	Physically Inactive	0.91	0.70 - 1.19	0.51	**1 (reference)**			0.99	0.79 - 1.23	0.90	**1 (reference)**		
	Moderate	Physically Active	0.80	0.60 - 1.06	0.11	0.86	0.65 - 1.15	0.31	0.87	0.70 - 1.08	0.22	0.88	0.71 - 1.11	0.28
	High	Physically Inactive	0.91	0.70 - 1.18	0.48	**1 (reference)**			1.03	0.82 - 1.28	0.83	**1 (reference)**		**--**
	High	Physically Active	0.73	0.55 - 0.97	2.95×10^{-2}	0.79	0.59 - 1.05	9.77×10^{-2}	0.71	0.57 - 0.88	2.03×10^{-3}	0.69	0.55 - 0.86	9.24×10^{-4}

Abbreviations: FA, Favourable Adiposity (‡ Estimated genetic liability without rs12369179, rs4684847, rs987469 and rs573454216).

a Adjusted for age, sex, smoking status, alcohol status, meat and fish intake, fruit and vegetable intake, low-density lipoprotein cholesterol, high-density lipoprotein, and diabetes (diagnosed by doctor OR using insulin medication OR glucose ≥ 7.0mmol/l OR HbA1C ≥ 48mmol/mol (6.5%).

b , c, *, Odds ratio, 95% confidence interval and p-value for odds ratio, are provided for the joint association between favourable adiposity genetic liability and physical activity status on the odds of hypertension compared with the reference group (Low genetic liability combined with physically Inactive). The values are derived from logistic regression models.

d , e, #, Odds ratio, 95% confidence interval and p-value for odds ratio, are provided for difference in physical activity status for each category of genetic liability on the odds of hypertension compared with the reference group (comprising of similar genetic liability combined with physically Inactive). The values are derived from logistic regression models.

Physical Activity was derived through median-split overall accelerometer data. The threshold for physically active participants was ≥ 27.61 milli-gravity

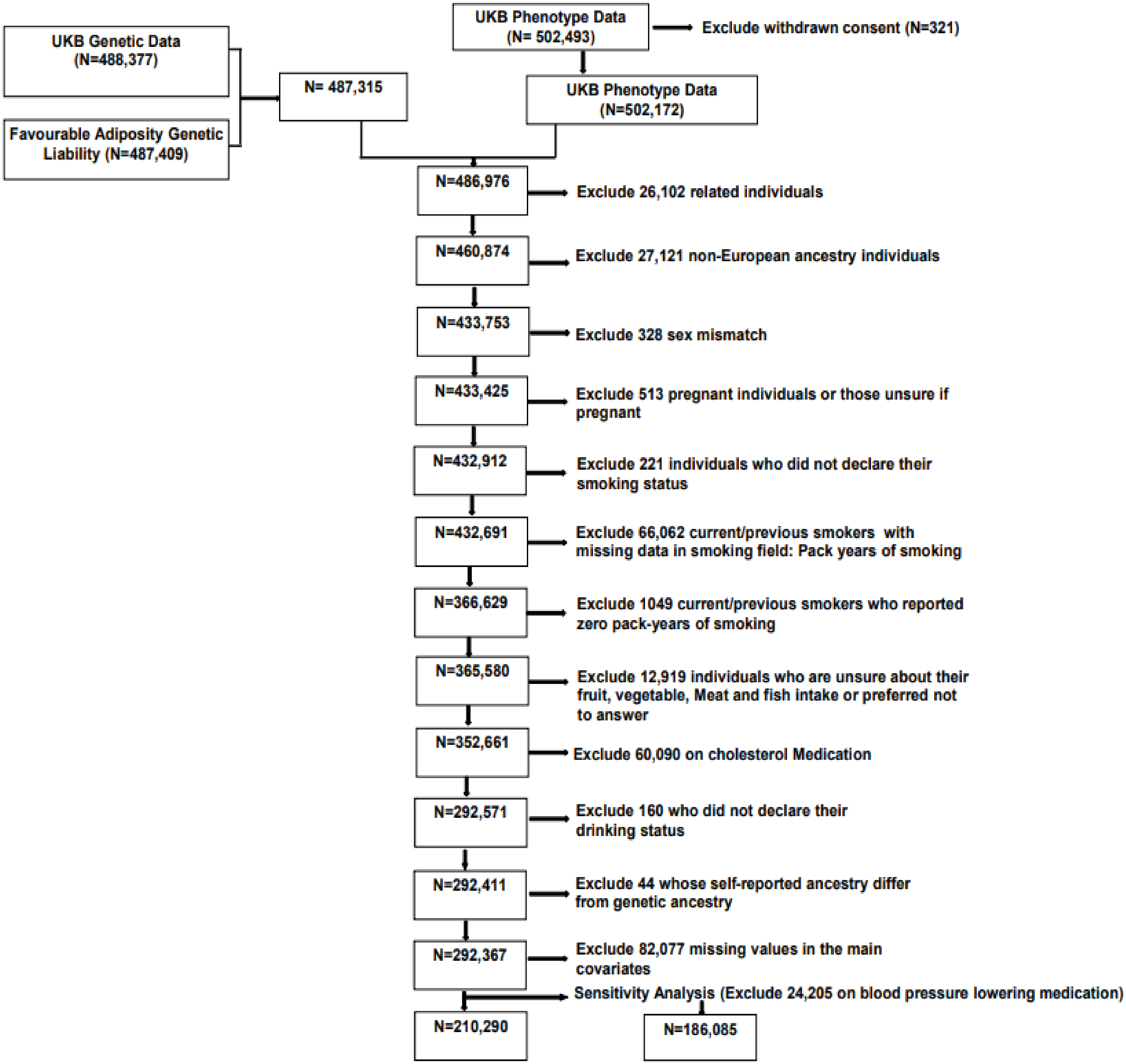

Figure S1. Flow chart of eligible population illustrating the phenotype and genotype quality control within the UK Biobank data.
